# Safety and Immunogenicity Following Administration of a Live, Attenuated Monovalent 2009 H1N1 Influenza Vaccine to Children and Adults in Two Randomized Controlled Trials

**DOI:** 10.1371/journal.pone.0013755

**Published:** 2010-10-29

**Authors:** Raburn M. Mallory, Elissa Malkin, Christopher S. Ambrose, Terramika Bellamy, Li Shi, Tingting Yi, Taff Jones, George Kemble, Filip Dubovsky

**Affiliations:** MedImmune, LLC, Gaithersburg, Maryland, United States of America; University of Sao Paulo, Brazil

## Abstract

**Background:**

The safety, tolerability, and immunogenicity of a monovalent intranasal 2009 A/H1N1 live attenuated influenza vaccine (LAIV) were evaluated in children and adults.

**Methods/Principal Findings:**

Two randomized, double-blind, placebo-controlled studies were completed in children (2–17 y) and adults (18–49 y). Subjects were assigned 4∶1 to receive 2 doses of H1N1 LAIV or placebo 28 days apart. The primary safety endpoint was fever ≥38.3°C during days 1–8 after the first dose; the primary immunogenicity endpoint was the proportion of subjects experiencing a postdose seroresponse. Solicited symptoms and adverse events were recorded for 14 days after each dose and safety data were collected for 180 days post-final dose. In total, 326 children (H1N1 LAIV, n = 261; placebo, n = 65) and 300 adults (H1N1 LAIV, n = 240; placebo, n = 60) were enrolled. After dose 1, fever ≥38.3°C occurred in 4 (1.5%) pediatric vaccine recipients and 1 (1.5%) placebo recipient (rate difference, 0%; 95% CI: –6.4%, 3.1%). No adults experienced fever following dose 1. Seroresponse rates in children (H1N1 LAIV vs. placebo) were 11.1% vs. 6.3% after dose 1 (rate difference, 4.8%; 95% CI: –9.6%, 13.8%) and 32.0% vs. 14.5% after dose 2 (rate difference, 17.5%; 95% CI: 5.5%, 27.1%). Seroresponse rates in adults were 6.1% vs. 0% (rate difference, 6.1%; 95% CI: –5.6%, 12.6%) and 14.9% vs. 5.6% (rate difference, 9.3%; 95% CI: –0.8%, 16.3%) after dose 1 and dose 2, respectively. Solicited symptoms after dose 1 (H1N1 LAIV vs. placebo) occurred in 37.5% vs. 32.3% of children and 41.7% vs. 31.7% of adults. Solicited symptoms occurred less frequently after dose 2 in adults and children. No vaccine-related serious adverse events occurred.

**Conclusions/Significance:**

In subjects aged 2 to 49 years, two doses of H1N1 LAIV have a safety and immunogenicity profile similar to other previously studied and efficacious formulations of seasonal trivalent LAIV.

**Trial Registration:**

ClinicalTrials.gov NCT00946101, NCT00945893

## Introduction

In response to the 2009 H1N1 influenza pandemic, MedImmune (Gaithersburg, MD) developed a live attenuated intranasal H1N1 vaccine based on the Ann Arbor 6∶2 reassortant technology used to produce the annual trivalent seasonal influenza vaccine (MedImmune, Gaithersburg, MD) [1], [2]. Live attenuated influenza vaccines (LAIVs) are well suited to confront pandemic and epidemic influenza and may confer distinct advantages compared with inactivated or subunit vaccines [3]. Data from 3 large placebo-controlled clinical studies indicate that relatively high levels of efficacy (ranging from 60% to 90%) are seen in previously unvaccinated young children after a single dose of trivalent LAIV. Efficacy following a single dose of LAIV is an important consideration for pandemic influenza; experiences with unadjuvanted, inactivated seasonal and H5N1 influenza vaccines indicate that two doses may be required in order to generate robust immune responses to novel influenza strains in unprimed individuals such as young children [4]–[6]. However, fewer than 25% of children 2 to 8 years of age who are recommended to receive two doses of seasonal influenza vaccines actually receive both doses [7]. LAIV has also demonstrated protection against influenza strains in children and adults that are antigenically distinct from those contained in the vaccine [8]–[11]. At the onset of the pandemic, potential protection against drifted strains was considered a useful attribute of the vaccine as it was not known to what extent circulating strains of H1N1 might antigenically drift from the vaccine strain over time. LAIV may also induce an innate antiviral state that results in protection from influenza during the days immediately after vaccination; this would clearly be relevant if high levels of influenza transmission were already present when the vaccine became available [12], [13]. Due to manufacturing capacity advantages, LAIVs may also be the preferred technology to address a pandemic [14]. Finally, administration of LAIV is rapid, needle-free, and avoids issues associated with blood exposure and use of sharps, and is thus well suited to mass vaccination campaigns in community settings such as schools [15]–[17].

The objective of the clinical studies was to evaluate the safety, tolerability, and immunogenicity of 2 doses of a monovalent intranasal A/H1N1 LAIV administered 28 days apart in children and adults prior to U.S. licensure and subsequent widespread distribution of the vaccine. Interim results were provided to the US Food and Drug Administration (FDA) as they became available; this report provides the complete long-term safety and immunogenicity data for the 2 studies.

## Methods

Full trial protocols of the two studies and the CONSORT checklist for this report are available as supporting information; see Protocol S1, Protocol S2, and Checklist S1, respectively.

### Ethics

Individual participants or their parents/legal representatives gave written informed consent. Pediatric assent was also obtained, if appropriate. The study protocol and consent/assent forms were approved by the Copernicus Group Institutional Review Board, Research Triangle Park, NC.

### Study Design

Two randomized, double-blind, placebo-controlled studies were conducted at multiple sites in the United States to evaluate the safety and immunogenicity of 2 doses of H1N1 LAIV in healthy children aged 2 to 17 years (Clinicaltrials.gov identifier, NCT00946101) and healthy adults aged 18 to 49 years (Clinicaltrials.gov identifier, NCT00945893). The design of the studies was modeled on studies that have been conducted annually in the United States to evaluate the attenuation of LAIV strains expressing updated hemagglutinin (HA) and neuraminidase (NA) antigens before their incorporation into trivalent seasonal LAIV formulations (e.g., Clinicaltrials.gov identifier, NCT00873912). Eligible subjects were randomly assigned using an interactive voice response system in a 4∶1 ratio to receive 2 doses of live monovalent H1N1 LAIV or placebo by intranasal spray 28 days apart (i.e., on days 1 and 29). In the adult study, randomization was stratified by site. In the pediatric study, randomization was stratified by age (2–8 y and 9–17 y). Subjects in both studies were further randomized (1∶1) to provide a blood sample on either day 15 or day 29 after their first vaccination. A final immunogenicity blood sample was collected on day 57, approximately 28 days after the second vaccination. After the blinded portion of the study was concluded, subjects randomized to receive placebo in the studies were offered optional H1N1 vaccination after collection of their Day 57 blood sample. The studies were conducted in compliance with the International Conference on Harmonisation Guidelines for Good Clinical Practice and the Declaration of Helsinki.

### Vaccine

The 2009 H1N1 LAIV was produced by MedImmune and was derived by genetic reassortment of the hemagglutinin and neuraminidase genes from the wild-type A/California/7/2009 virus and the remaining 6 gene segments from an attenuated master donor virus as previously described [1], [2], [18]. The resulting 6∶2 reassortant vaccine virus is a temperature-sensitive, cold-adapted, attenuated virus that is grown in chicken eggs using the same manufacturing process used to produce MedImmune's seasonal trivalent LAIV. Monovalent vaccine was supplied in intranasal spray applicators containing approximately 10^7^ fluorescent focus units (FFU) of the reassortant influenza virus in a total volume of 0.5 mL of sucrose-phosphate buffer and egg allantoic fluid (0.25 mL administered into each nostril). Placebo (0.5 mL of sucrose-phosphate buffer) was supplied and administered using identical intranasal applicators.

### Subjects

Exclusion criteria included hypersensitivity to any component of the vaccine or placebo; medical conditions that predispose to complications from influenza (e.g. lung disease, heart disease, renal disease, metabolic disease such as diabetes); acute febrile and/or clinically significant respiratory illness within 14 days of randomization; history of asthma, recurrent wheezing (in children <5 years of age), or history of Guillain-Barré syndrome; or any known immunosuppressive condition or immune deficiency disease. All women of child-bearing potential were required to have a negative pregnancy test at screening and immediately before each vaccination. Complete eligibility criteria are described in Supporting Text S1.

### Safety Assessments

The primary safety analysis compared the rates of fever during days 1 to 8 after dose 1. Fever was defined as a temperature ≥38.3°C (101°F). Additional safety endpoints included solicited symptoms, adverse events (AEs), and antipyretic and analgesic use from day 1 through day 8 and from day 1 through day 15 after each vaccination. Serious adverse events (SAEs) and new onset chronic diseases (NOCDs) were collected through 180 days after the final dose. Memory aid worksheets were provided to record solicited symptoms, AEs, and concomitant medication use for 14 days after dosing. Solicited symptoms, reported as present or absent, included fever (temperature was recorded daily), runny nose (adults) or runny/stuffy nose (children), sore throat, cough, vomiting (adults), muscle aches, chills (adults), decreased activity, decreased appetite (children), and headache. Antipyretic and/or analgesic use was discouraged during the 14 days postvaccination to avoid masking the primary safety endpoint of fever. Subjects who experienced a febrile illness within 7 days after dose 1 were instructed to return to the study site for evaluation.

### Laboratory Assays

To assess humoral responses to the vaccine, serum antibody titers were measured at randomization (baseline) and on day 15 or 29 after dose 1 and on day 57 (28 days after dose 2) using a standardized hemagglutination inhibition (HAI) assay against antigenically matched influenza A/H1N1 6∶2 virus reassortants, performed as previously described [19]. Full details are provided in Supporting Text S2.

### Statistical Analyses

Sample size and power calculations were based on the primary safety endpoint. A sample size of 300 was estimated to provide at least 80% power to detect a 10 percentage point difference in the rate of fever in children if the true difference was less than 1% and the true fever rate in the vaccine group was less than 8%. The same sample size (n = 300) was estimated to provide at least 99% power to rule out a fever rate difference of 10 percentage points in adults if the true difference was 0% and the true fever rate in the vaccine group was less than 3%. A 2-sided 95% exact confidence interval (CI) for the rate difference (vaccine minus placebo) was calculated based on score statistics proposed by Chan and Zhang [20] and the upper limit of the CI was evaluated against a pre-specified equivalence criteria of 10%. Rate differences and the exact 2-sided 95% CIs for the rate differences were also calculated for other reported solicited symptoms between the two treatment groups; there were no pre-specified equivalence criteria for these secondary analyses. The incidence of AEs and the proportion of subjects using antipyretics and/or analgesics on days 1 to 8 and days 1 to 15 after doses 1 and 2 were also summarized.

The primary immunogenicity endpoint was the proportion of subjects experiencing a postvaccination seroresponse in baseline seronegative subjects and in all subjects regardless of baseline serostatus. Seroresponse was defined as a ≥4-fold rise in HAI titer from baseline. Subjects with baseline HAI titers of ≤4 were considered seronegative. Secondary immunogenicity endpoints were the proportion of subjects with a postdose HAI titer ≥32 and HAI geometric mean titers (GMTs). Two-sided exact 95% CIs were constructed for rate differences using the exact method proposed by Chan and Zhang [20]. Geometric mean titers (GMTs) were calculated as GMT = anti-log_e_(mean[log_e_ X_1i_]), where X_1i_ is the postdose assay result for subject i.

The intent-to-treat (ITT) population included all randomized subjects based on treatment assignment. All subjects who received at least 1 dose of study vaccine and had any safety follow-up comprised the safety population. Subjects were considered part of the post dose 1 immunogenicity population if they received dose 1 of the study vaccine and had valid HAI measurements from blood samples obtained at baseline and post dose 1. Subjects were considered part of the post dose 2 immunogenicity population if they received 2 doses of the same study vaccine and had valid HAI measurements from blood samples obtained at baseline and post dose 2. Subjects with major protocol violations were not included in the immunogenicity populations.

## Results

### Subjects

**Figure 1:**
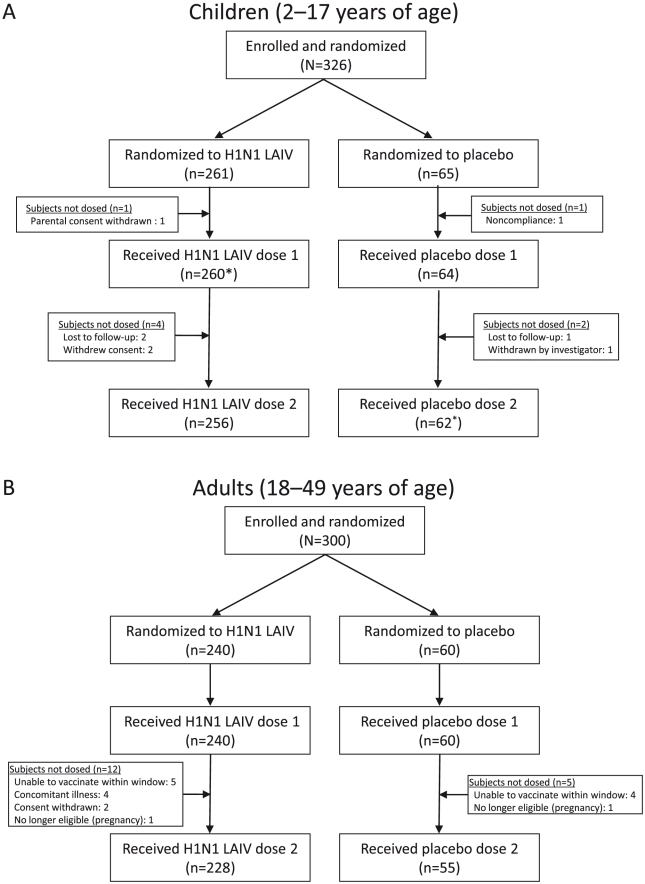
Subject Disposition (ITT Population): (A) Children and (B) Adults. ITT = intent to treat; LAIV = live attenuated influenza vaccine. *One child randomized to receive H1N1 LAIV was inadvertently administered placebo for dose 1; this subject also received placebo for dose 2. This subject was included in the H1N1 group for ITT analyses, but was grouped with placebo subjects for safety analyses.

From August 17–19, 2009, a total of 326 children and 300 adults were randomized at a 4∶1 ratio to receive H1N1 LAIV or placebo (**Figure 1**). Demographic characteristics of the ITT populations are summarized in **Table 1**. The mean ages for children were 8.9 and 9.2 years for the HINI LAIV and placebo groups, respectively and for adults were 33.3 and 34.1 years, respectively. Of the 326 randomized children, 324 subjects received dose 1 and were included in the safety analyses (H1N1 LAIV, n = 259; placebo, n = 65), and 319 subjects (H1N1 LAIV, n = 256, placebo, n = 63) received dose 2. One child randomized to receive H1N1 LAIV inadvertently received placebo at dose 1 and was included in the H1N1 LAIV group in the ITT population, but was included among placebo recipients for dose 1 safety analyses. All 300 randomized adults received dose 1 (H1N1 LAIV, n = 240; placebo, n = 60), and 283 received dose 2 (H1N1 LAIV, n = 228; placebo, n = 55).

**Table 1:** Demographics by Treatment Group (Intent-to-Treat Population).

	Children (2–17 y)		Adults (18–49 y)	
Baseline Characteristics	H1N1 LAIV	Placebo	H1N1 LAIV	Placebo
n	261	65	240	60
Age, y				
Mean (SD)	8.9 (4.3)	9.2 (4.3)	33.3 (9.2)	34.1 (8.9)
Median	8.0	10.0	33.0	32.0
Minimum–maximum	2–17	2–17	18–49	18–49
Age group, n (%)				
2–8 y	133 (51.0)	31 (47.7)	NA	NA
9–17 y	128 (49.0)	34 (52.3)	NA	NA
Gender, n (%)				
Male	131 (50.2)	29 (44.6)	102 (42.5)	27 (45.0)
Female	130 (49.8)	36 (55.4)	138 (57.5)	33 (55.0)
Ethnicity, n (%)				
Hispanic or Latino	50 (19.2)	17 (26.2)	95 (39.6)	18 (30.0)
Non–Hispanic or –Latino	211 (80.8)	48 (73.8)	145 (60.4)	42 (70.0)
Race, n (%)				
American Indian or Alaskan Native	4 (1.5)	0 (0.0)	1 (0.4)	0 (0.0)
Asian	2 (0.8)	4 (6.2)	0 (0.0)	0 (0.0)
Black or African American	43 (16.5)	12 (18.5)	37 (15.4)	13 (21.7)
Native Hawaiian or Pacific Islander	2 (0.8)	0 (0.0)	1 (0.4)	0 (0.0)
White	198 (75.9)	43 (66.2)	199 (82.9)	47 (78.3)
Other	4 (1.5)	5 (7.7)	1 (0.4)	0 (0.0)
Multiracial	8 (3.1)	1 (1.5)	1 (0.4)	0 (0.0)

LAIV = live attenuated influenza vaccine; NA = not applicable.

### Safety Analyses

Safety data was collected from 324 children (H1N1 LAIV, n = 259; placebo, n = 65) after dose 1 and 318 children (H1N1 LAIV, n = 255; placebo, n = 63) after dose 2, and from 300 adults (H1N1 LAIV, n = 240; placebo, n = 60) after dose 1 and 283 adults (H1N1 LAIV, n = 228; placebo, n = 55) after dose 2. There was no statistical difference between treatment groups for the primary endpoint (fever ≥38.3°C for days 1–8 postdose 1) in children or adults. Among children, fever ≥38.3°C occurred in 1.5% (n = 4) of H1N1 LAIV and 1.5% (n = 1) of placebo recipients after dose 1 (rate difference, 0%; 95% CI: –6.4%, 3.1%) and 1.2% (n = 3) and 0% after dose 2 (rate difference, 1.2%; 95% CI: –4.1%, 3.7%). Fever was not reported among adult subjects after dose 1 but was reported in 0.4% (n = 1) and 1.8% (n = 1) of H1N1 LAIV and placebo recipients after dose 2 (rate difference, –1.4%; 95% CI: –8.7%, 1.4%). In both children and adults, antipyretic and/or analgesic use following dose 1 and 2 was not significantly different among H1N1 LAIV and placebo recipients (data not shown).

**Figure 2:**
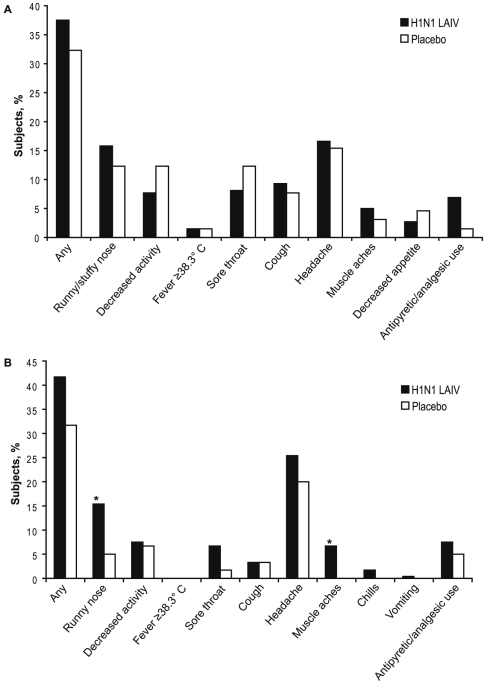
Solicited Symptoms in (A) Children and (B) Adults Through Day 8 Postvaccination with Dose 1. *P<0.05.

**Figure 3:**
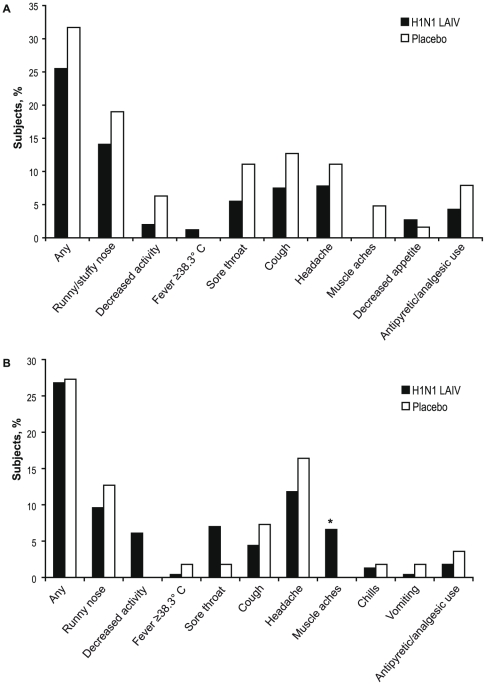
Solicited Symptoms in (A) Children and (B) Adults Through Day 8 Postvaccination with Dose 2. *P<0.05.

Solicited symptoms were collected in children and adults from day 1 through day 15 after both doses. Data collected through day 8 post dose 1 and dose 2 are presented in **Figures 2** **and** **3** (data through day 15 after each dose are presented in **Supplemental Figures S1 and S2**). In general, H1N1 LAIV recipients reported more solicited symptoms compared with placebo recipients. Through day 8 after dose 1, 37.1% of children receiving H1N1 LAIV and 32.3% of placebo children reported at least one solicited symptom (rate difference 4.8%; 95% CI: –8.%, 17.2%); among adults, the percentages were 41.7% and 31.7%, respectively (rate difference, 10.0%; 95% CI: –4.1%, 22.8%; **Figure 2**). The percentage of individuals reporting solicited symptoms decreased in both adults and children after dose 2 (**Figure 3**).

The most common solicited symptom in children receiving H1N1 LAIV through day 8 post dose 1 was headache which was reported by 16.6% and 15.4% of H1N1 LAIV and placebo recipients, respectively (rate difference, 1.2%; 95% CI: –10.2%, 10.2%) The most common solicited symptom through day 8 post dose 2 in children receiving H1N1 LAIV was runny/stuffy nose (Figure 2). For children, the rate differences for all solicited symptoms were not statistically significant between H1N1 LAIV and placebo recipients.

In adults, the most common solicited symptom reported through day 8 post dose 1 was headache which was reported by 25.4% and 20.0% of H1N1 LAIV and placebo recipients, respectively, after dose 1 and 11.8% and 16.4% through day 8 after dose 2. The rate of headaches in adults receiving H1N1 LAIV compared with placebo did not differ significantly. Through day 8 after dose 1 significantly more adults who received H1N1 LAIV experienced runny nose (H1N1 LAIV, 15.4%; placebo 5.0% [rate difference, 10.4%; 95% CI: 1.2%,17.2%]) and muscle aches (H1N1 LAIV, 6.7%; placebo, 0.0%; [rate difference, 6.7%; 95% CI: 0.8%,10.8%]). For adults receiving H1N1 LAIV the incidence of solicited symptoms was lower through day 8 following dose 2 and only muscle aches were significantly greater in H1N1 LAIV recipients. No other rate differences for solicited symptoms in adults were significant.

Adverse events (AEs) were collected during days 1–15 after doses 1 and 2 in children and adults. In children, AEs after dose 1 were reported in 18.1% and 16.9% of H1N1 LAIV and placebo recipients, respectively, and in 13.7% and 14.3% of recipients after dose 2 (**Supplemental Table S1**). The most common AEs in children after dose 1 were nausea (1.9% vs 3.1%), vomiting (2.7% vs 1.5%), and diarrhea (1.5% vs 1.5%). The overall frequency of adverse events was lower following dose 2. Three SAEs were reported in children during the study, hospitalization for depression and osteomyelitis in vaccine recipients and cellulitis in a placebo recipient; all were considered unrelated to study vaccine. One new onset chronic disease (NOCD), attention deficit hyperactivity disorder, was reported in a placebo recipient.

In adults, AEs were reported by 15.8% of H1N1 LAIV recipients and 16.7% of placebo recipients after dose 1 and 7.9% and 7.3% after dose 2 (**Supplemental Table S2**). Generally, more AEs were reported after dose 1 and the most common were nausea (H1N1 LAIV, 2.1%; placebo, 3.3%), nasal congestion (1.7% vs 1.7%), and sneezing (1.7% vs 3.3%). Four SAEs were reported during the study in adults for cellulitis and depression in vaccine recipients, gallbladder disease and possible cervical cancer in placebo recipients; all were considered unrelated to study vaccine. Two NOCDs, hypothyroidism in a vaccine recipient and possible cervical cancer in a placebo recipient were reported and not considered to be treatment-related. One adult subject who received H1N1 LAIV was diagnosed with A/H1N1 influenza 13 days after dose 1 and was discontinued from the study.

### Immunogenicity

Serum for HAI antibody titer analysis was collected at baseline, on day 15 or 29 after dose 1 and on day 57 (28 days after dose 2) in both children and adults. The proportions of H1N1 LAIV and placebo recipients who were seronegative at baseline were comparable for children and adults (children: H1N1 LAIV, 88.6%; placebo, 90.6%; adults: H1N1 LAIV, 85.1%; placebo, 76.3%). Among all children regardless of baseline serostatus, seroconversion rates after vaccination with H1N1 LAIV were 7.8% and 11.1% for study days 15 and 29, respectively, and 32.0% on day 57. For placebo recipients, seroconversion rates were 6.3% on days 15 and 29, and 14.5% on day 57 (**Table 2**). For the subset of all children 2 to 9 years of age (regardless of baseline serostatus) who are recommended to receive two doses of the H1N1 vaccine [21] seroconversion rates were similar; 8.5%, 15.1% and 28.0% for vaccine recipients and 0%, 0% and 6.7% for placebo recipients on days 15, 29 and 57, respectively (**Table 2**). Among adults regardless of baseline serostatus, seroconversion rates after H1N1 LAIV were 2.5% and 6.1% for days 15 and 29, respectively, and 14.9% on day 57. For adult placebo recipients regardless of baseline serostatus, seroconversion rates were 0% on day 15 and 29 and 5.6% on day 57 (**Table 3**). Seroconversion rates were slightly higher among adult subjects who were seronegative at baseline.

**Table 2:** Immunogenicity Data for Children 2–17 years.

	Baseline seronegative				All recipients				Children aged 2–9 years			
	Day 0n = 226, 58*	Day 15n = 112, 28*	Day 29n = 114, 30*	Day 57n = 221, 56*	Day 0n = 255, 64*	Day 15n = 129, 32*	Day 29n = 126, 32*	Day 57n = 250, 62*	Day 0n = 144, 30*	Day 15n = 71, 16*	Day 29n = 73, 14*	Day 57n = 143, 30
GMT												
Vaccine	2.03	2.53	2.65	6.05	2.81	3.55	3.53	7.61	2.48	3.15	3.42	6.20
Placebo	2.01	2.21	2.35	3.08	2.50	2.89	2.71	3.70	2.64	3.08	2.32	3.10
Seroconversion rate, %												
Vaccine	NA	8.9	11.4	34.8	NA	7.8	11.1	32.0	NA	8.5	15.1	28.0
Placebo	NA	7.1	6.7	16.1	NA	6.3	6.3	14.5	NA	0	0	6.7
GMT ≥32, n (%)												
Vaccine				42 (19.0)				66 (26.4)				33 (23.1)
Placebo				4 (7.1)				6 (9.7)				2 (6.7)
Rate difference, % (95% CI)				11.9(11.3, 19.6)				16.7(5.9, 25.2)				16.4(0.7, 26.6)

GMT = geometric mean titer; NA = not applicable.

*All n's are presented as vaccine, placebo.

**Table 3:** Immunogenicity Data for Adults.

		**Baseline seronegative**				**All recipients**			
		**Day 0** **n = 200, 45** *	**Day 15** **n = 101, 26** *	**Day 29** **n = 99, 19** *	**Day 57** **n = 189, 42** *	**Day 0** **n = 235, 59** *	**Day 15** **n = 120, 30** *	**Day 29** **n = 115, 29** *	**Day 57** **n = 222, 54** *
GMT									
	Vaccine	2.15	2.42	2.57	3.65	3.00	3.46	3.44	4.86
	Placebo	2.09	2.00	2.31	2.48	3.64	2.64	4.96	3.90
Seroconversion rate,%									
	Vaccine	NA	3.0	7.1	16.9	NA	2.5	6.1	14.9
	Placebo	NA	0.0	0.0	7.1	NA	0.0	0.0	5.6
GMT ≥32, n (%)									
	Vaccine				14 (7.4)				30 (13.5)
	Placebo				1 (2.4)				6 (11.1)
	Rate difference, % , (95% CI)				5.0(–4.9, 10.7)				2.4(–9.3, 10.8)

GMFR = geometric mean fold rise; GMT = geometric mean titer; NA = not applicable.

*All n’s are presented as vaccine, placebo.

## Discussion

The 2009 H1N1 LAIV vaccine, administered as 2 doses 28 days apart, has a reassuring safety profile and is well tolerated in children and adults. The local and systemic symptoms observed in these studies are consistent with intranasal viral replication, are comparable to what has been observed in previous studies with seasonal LAIV, and demonstrate that this H1N1 LAIV strain is appropriately attenuated. The overall safety profile of the vaccine is consistent with that reported for other seasonal LAIV vaccines [10], [22]–[26], which is expected since these vaccines are generated from the same master donor virus responsible for conferring attenuation, cold-adaptation and temperature sensitivity [27]. While the number of subjects enrolled in these studies would not have allowed for the detection of rare safety signals, the overall safety of the vaccine is supported by post-marketing surveillance data [28]. The U.S. Centers for Disease Control and Prevention analyzed adverse events received through the Vaccine Adverse Event Reporting System (VAERS) and electronic data available from the Vaccine Safety Datalink, a population-based database that included over 400,000 persons who received H1N1 vaccinations. The analysis covered the period of October 1 to November 24, 2009, during which approximately 11.3 million doses of the live attenuated influenza vaccine were distributed, and showed no concerning safety signals (i.e., new, unexpected, or rare adverse events) and no increased occurrence of monitored conditions, including Guillain-Barré syndrome.

The seroconversion rates after H1N1 LAIV vaccination observed in these studies are consistent with those previously reported from clinical trials conducted with MedImmune's seasonal trivalent LAIV [22], [24], [25], [29]–[34]. A general trend observed in trivalent LAIV studies is that adults demonstrate limited HAI responses to LAIV whereas young children, particularly those without pre-existing antibodies, can exhibit higher rates of seroconversion in response to vaccination. In adults, strain specific seroresponse rates ranging from 4% to 40% have previously been reported following a single dose of LAIV [29], [35], while in children responses after 2 doses of LAIV have been more variable, ranging from 33% to >90% in seronegative children and from 22% to approximately 90% in all children regardless of baseline serostatus [22], [24], [25], [29]–[34]. The seroresponse rates observed in placebo recipients in both studies is likely attributable to 2009 H1N1 infections occurring in the U.S. during the period in which these studies were conducted.

The measurement of serum HAI responses following administration of LAIV represents a biologically-relevant strain-specific functional immune response. Serum HAI responses are a useful biomarker to assess comparability of immune responses and have enabled previous assessments of formulation bridging, manufacturing and lot consistency, as well as concomitant administration of seasonal LAIV with other live virus vaccines. However for LAIV, HAI titers are not well correlated with protection against influenza-like illness since studies have shown vaccine efficacy in the absence of high rates of serum HAI antibody response [35], [36]. In the 6 placebo-controlled pediatric efficacy studies that have been conducted with LAIV [37], the seroresponse rate for all subjects for A/H1N1 strains has ranged from 28% to 60%. In five of these studies clinically significant efficacy was demonstrated against circulating H1N1 strains matched to the vaccine (ranging from 81% to 100%) while in the sixth study A/H1N1 strains did not circulate in the community which precluded an estimate of efficacy. Similarly, in a study of adults immunized with LAIV and subsequently challenged with strain-matched wild-type influenza viruses, the overall HAI seroconversion rate was 20% while efficacy against laboratory-documented influenza illness was 85% [35]. While direct information about the efficacy of the live attenuated 2009 H1N1 vaccine is not yet available, preliminary data are available from a community-based, open-label, non-randomized study of a school-located vaccination campaign in Texas in which 90% of the H1N1 vaccine administered to children between the ages of 4 and 18 years was LAIV [38]. The study compared the rates of febrile medically-attended acute respiratory illness due to influenza in intervention and comparison cities during the pandemic outbreak in central Texas (September 23 to December 12, 2009) and demonstrated statistically significant effectiveness in school-aged children 4 to 18 years of age (relative risk (RR), 0.70; 95% CI: 0.60, 0.81) and also demonstrated indirect effectiveness in adults 19 to 49 years of age (RR, 0.78; 95% CI: 0.69, 0.88).

These studies were designed to rapidly provide the US FDA with sufficient information to guide licensure decisions regarding MedImmune's monovalent live attenuated 2009 H1N1 vaccine in the setting of a widespread H1N1 pandemic; as a result they have a number of limitations. As mentioned previously, due the relatively small size of the studies they would not have been able to detect rare safety signals; however, the very rapid assessment and release of data from post-marketing surveillance has helped to address this issue. Additionally, these studies evaluated serum HAI antibody responses and did not evaluate vaccine replication or other aspects of the immune response to LAIV. The studies included HAI testing based on guidance from the US FDA for consistency with inactivated H1N1 vaccine studies.

These studies demonstrate that 2 doses of 2009 H1N1 LAIV are safe in healthy children and adults 2 to 49 years of age. Overall, the frequency of solicited symptoms and AEs were similar between H1N1 LAIV and placebo recipients, and most were mild to moderate in severity. While serum immune responses measured by HAI antibodies are are modest compared to levels achieved by inactivated vaccines, levels of these antibodies have not been shown to correlate well with protection against influenza for LAIV. Antibody levels seen in these studies are consistent with those reported in other clinical studies of LAIV in which clinically significant protection against influenza illness has been demonstrated.

## Supporting Information

Figure S1 Solicited Symptoms in (A) Children and (B) Adults Through Day 15 Postvaccination with Dose 1.
(0.06 MB PDF)

Figure S2 Solicited Symptoms in (A) Children and (B) Adults Through Day 15 Postvaccination with Dose 2.
(0.06 MB PDF)

Text S1 Inclusion Criteria.
(0.03 MB DOC)

Text S2 Laboratory Assays.
(0.02 MB DOC)

Table S1 Adverse Events Reported in Children ≤15 Days After Dose (Safety Population).
(0.04 MB DOC)

Table S2 Adverse Events Reported in Adults ≤15 Days After Dose (Safety Population).
(0.04 MB DOC)

Protocol S1 Pediatric Study MI-CP217.
(1.75 MB PDF)

Protocol S2 Adult Study MI-CP215.
(1.74 MB PDF)

Checklist S1 CONSORT Checklist.
(0.22 MB DOC)
