# Supplementary figures and images for: Safety and Immunogenicity Following Administration of a Live, Attenuated Monovalent 2009 H1N1 Influenza Vaccine to Children and Adults in Two Randomized Controlled Trials
Source: PLoS One. 2010 Oct 29;5(10):e13755. doi: 10.1371/journal.pone.0013755 (PMC2966412; doi:10.1371/journal.pone.0013755)

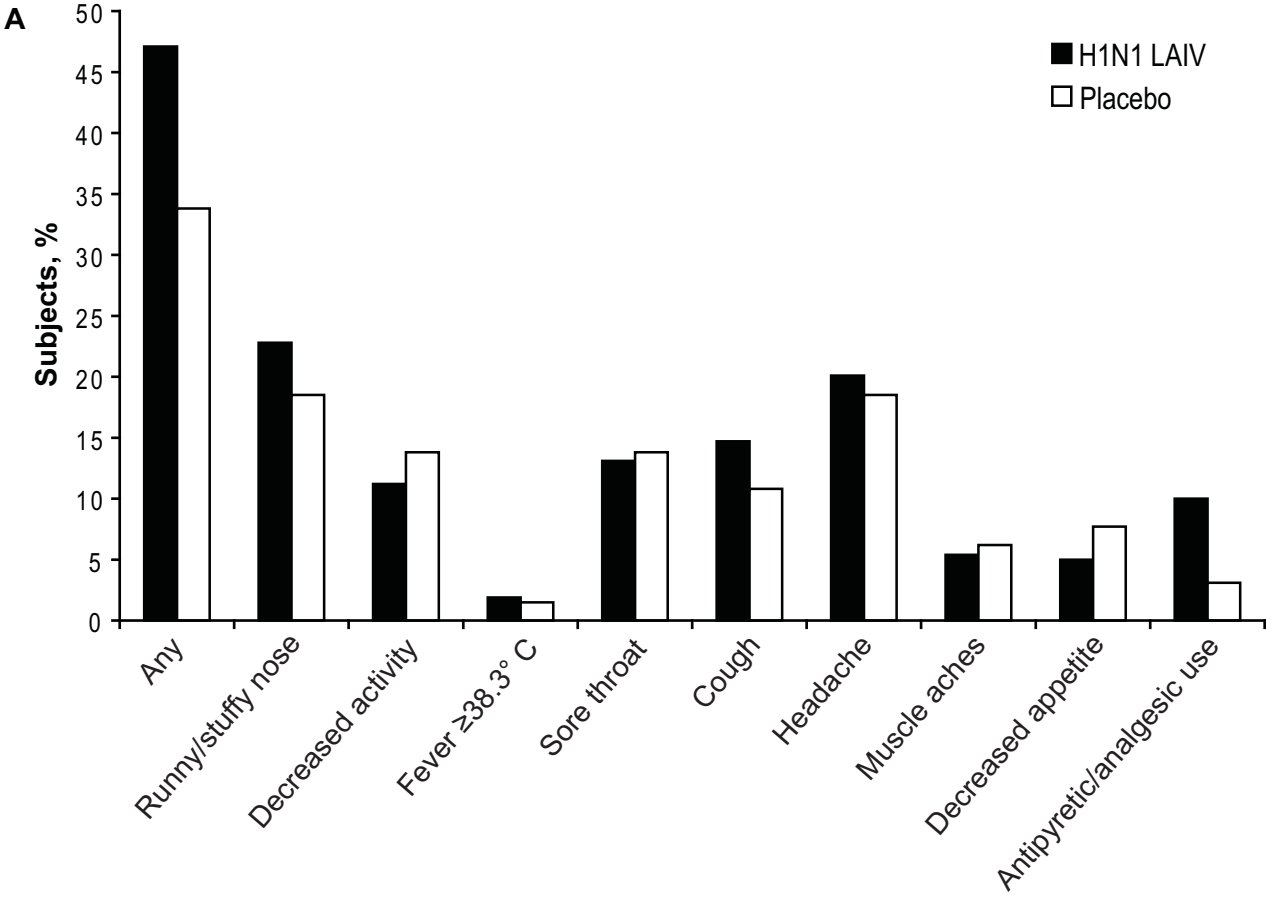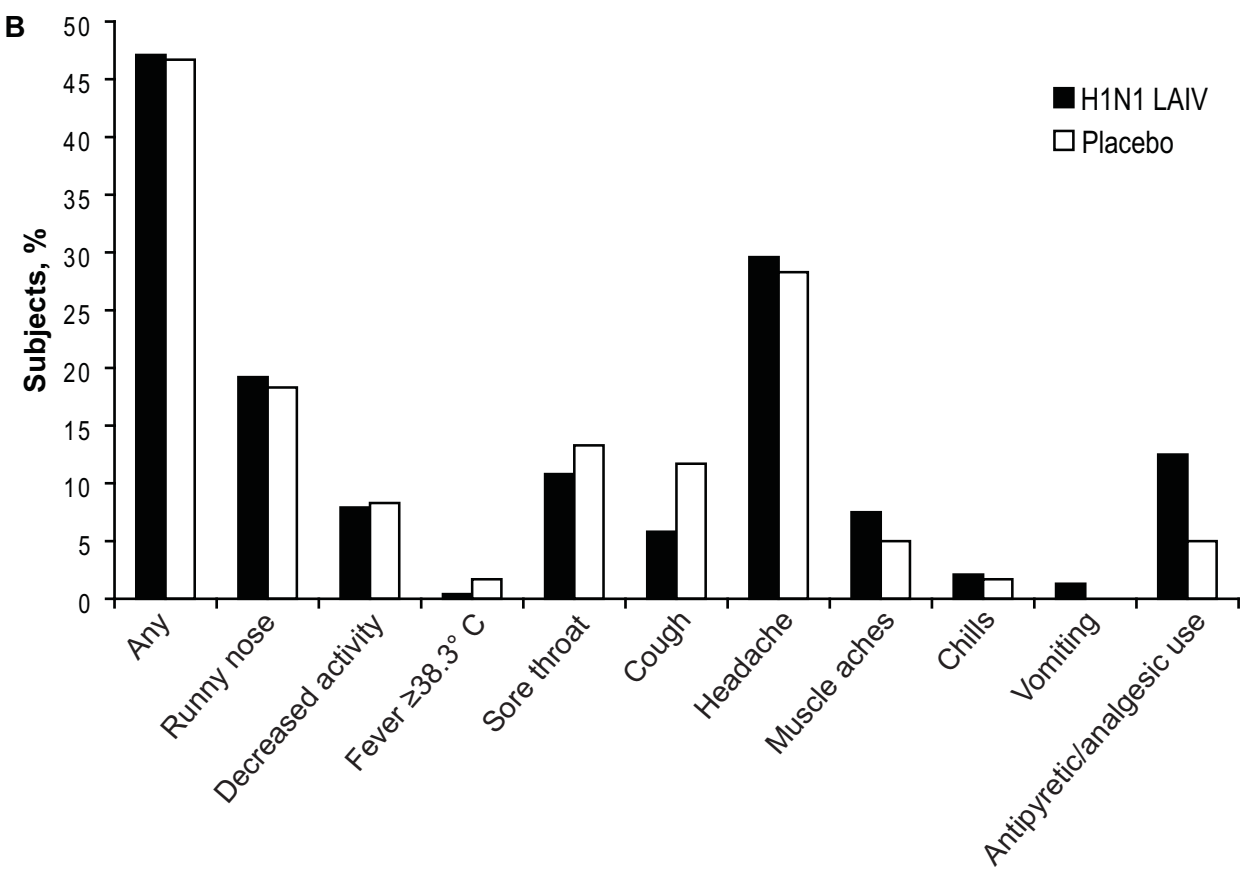

Supplement: Figure S1 — Solicited Symptoms in (A) Children and (B) Adults Through Day 15 Postvaccination with Dose 1. (0.06 MB PDF) [file pone.0013755.s001.pdf]

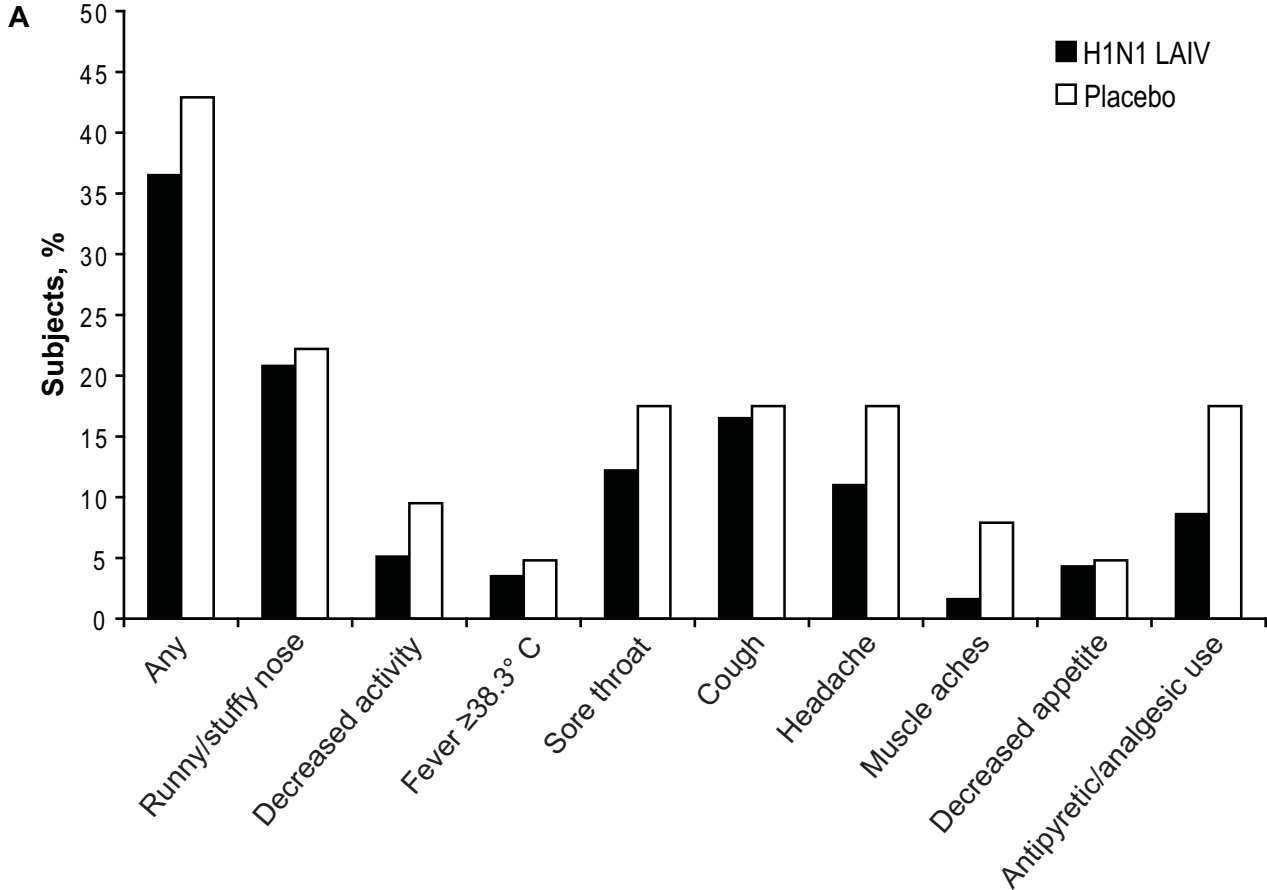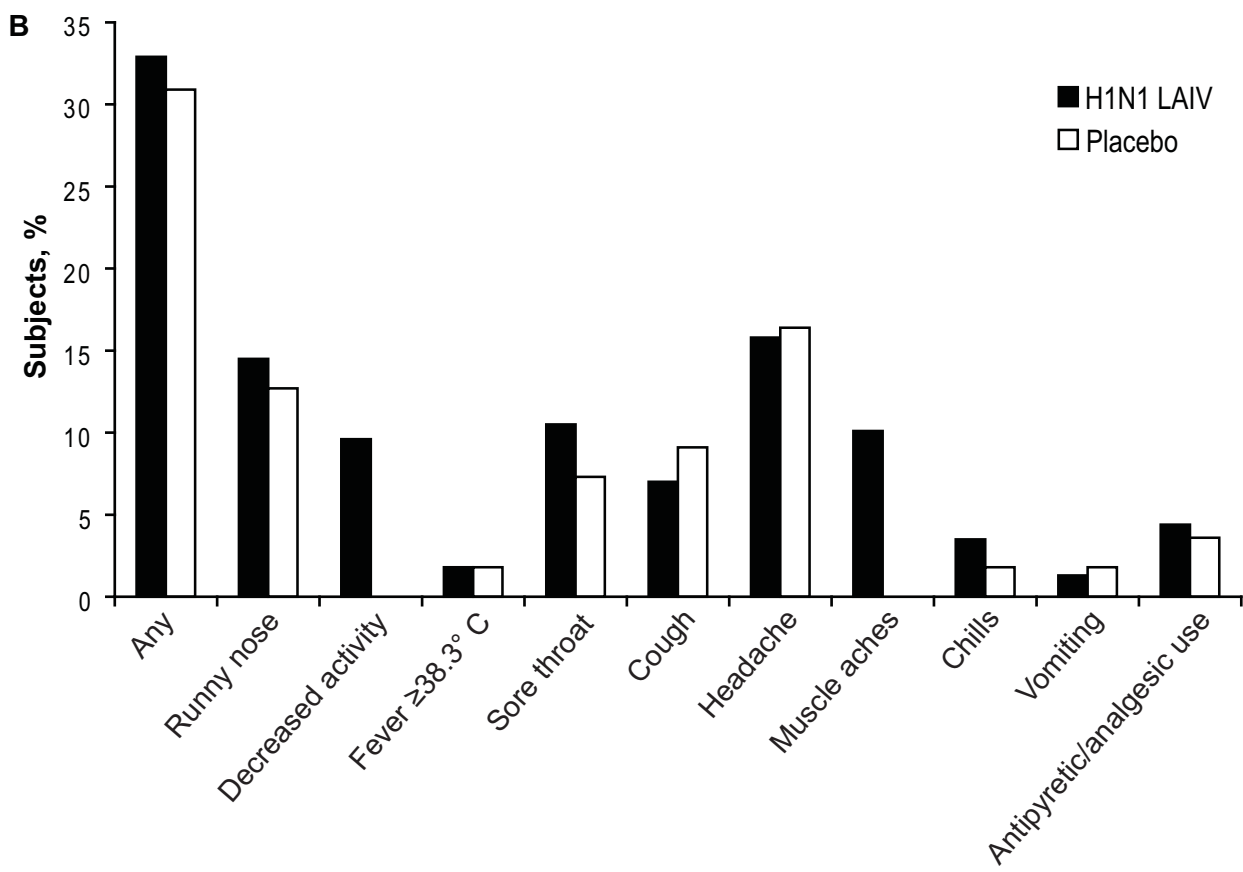

Supplement: Figure S2 — Solicited Symptoms in (A) Children and (B) Adults Through Day 15 Postvaccination with Dose 2. (0.06 MB PDF) [file pone.0013755.s002.pdf]
