# Supplementary material for: Safety and Immunogenicity Following Administration of a Live, Attenuated Monovalent 2009 H1N1 Influenza Vaccine to Children and Adults in Two Randomized Controlled Trials
Source: PLoS One. 2010 Oct 29;5(10):e13755. doi: 10.1371/journal.pone.0013755 (PMC2966412; doi:10.1371/journal.pone.0013755)
Supplement: Text S1 — Inclusion Criteria. (0.03 MB DOC) [file pone.0013755.s003.doc]

**Inclusion Criteria**

1. Male or female, aged 2 to 17 years (children) or 18 to 49 years (adults) at the time of randomization
2. Healthy by medical history and physical exam
3. Written informed consent and any locally required authorization (eg, HIPAA in the USA, EU Data Privacy Directive in the EU and written informed assent) obtained from the subject or their legal representative before performing any protocol-related procedures, including screening evaluations
4. Females of child-bearing potential, must use an effective method of avoiding pregnancy for 30 days before the first dose of investigational product, and must agree to continue using such precautions for 60 days after the second dose. In addition, the subject must also have a negative urine or blood pregnancy test at screening and, if screening and day 1 do not occur on the same day, on the day of vaccination before randomization. Investigator judgment is required to assess the childbearing potential of a pre-adolescent or adolescent girl.
5. Males, unless not sexually active, must use an effective method of birth control with a female partner and must agree to continue using such contraceptive precautions for at least 30 days after the second dose of investigational product (from day 1 through day 59 of the study)
6. Subject or their legal representative available by telephone
7. Subject or subject’s legal representative is able to understand and comply with the requirements of the protocol, as judged by the investigator
8. Ability to complete follow-up period of 180 days after dose 2 as required by the protocol

**Exclusion Criteria**

1. History of hypersensitivity to any component of the investigational product including egg or egg protein, gelatin or arginine, or serious, life-threatening, or severe reactions to previous influenza vaccinations
2. History of hypersensitivity to gentamicin
3. Any condition for which the inactivated influenza vaccine is indicated, including chronic disorders of the pulmonary or cardiovascular systems (eg, asthma), chronic metabolic diseases (eg, diabetes mellitus), renal dysfunction, or hemoglobinopathies that required regular medical follow-up or hospitalization during the preceding year
4. Acute febrile (> 100.0°F oral or equivalent) and/or clinically significant respiratory illness (eg, cough or sore throat) within 14 days before randomization
5. History of asthma, or history of recurrent wheezing in children aged <5 years
6. Any known immunosuppressive condition or immune deficiency disease, including HIV infection, or ongoing immunosuppressive therapy
7. History of Guillain-Barré syndrome
8. A household contact who is severely immunocompromised (eg, hematopoietic stem cell transplant recipient, during those periods in which the immunocompromised individual requires care in a protective environment); subject should additionally avoid close contact with severely immunocompromised individuals for at least 21 days after receipt of investigational product
9. Receipt of any investigational agent within 30 days before randomization, or expected receipt through 30 days after the second dose of investigational product (use of licensed agents for indications not listed in the package insert is permitted)
10. Use of aspirin or salicylate-containing products in children within 30 days before randomization or expected receipt through 30 days after final vaccination
11. Expected receipt of antipyretic or analgesic medication (non-salicylate-containing) on a daily or every other day basis from randomization through 14 days after receipt of each dose of investigational product
12. Administration of intranasal medications within 14 days before randomization, or expected receipt through 14 days after administration of each dose of investigational product
13. Receipt of any nonstudy vaccine within 30 days before or after dose 1 or expected receipt of any nonstudy vaccine within 30 days before or after dose 2.
14. Known or suspected mitochondrial encephalomyopathy
15. Subject is pregnant or a nursing mother
16. Any condition (eg, chronic cough, allergic rhinitis) that, in the opinion of the investigator, would interfere with evaluation of the investigational product or interpretation of subject safety or study results
17. Subject, legal representative, or immediate family member of subject is an employee of the clinical study site or is otherwise involved with the conduct of the study
