# Supplementary material for: Safety and Immunogenicity Following Administration of a Live, Attenuated Monovalent 2009 H1N1 Influenza Vaccine to Children and Adults in Two Randomized Controlled Trials
Source: PLoS One. 2010 Oct 29;5(10):e13755. doi: 10.1371/journal.pone.0013755 (PMC2966412; doi:10.1371/journal.pone.0013755)
Supplement: Text S2 — Laboratory Assays. (0.02 MB DOC) [file pone.0013755.s004.doc]

**Laboratory Assays**

Serum was separated and stored frozen at –20°C until used. Serum was treated with receptor-destroying enzyme (RDE) to remove nonspecific inhibitors of agglutination followed by adsorption to turkey erythrocytes to remove nonspecific agglutinins. Serial 2-fold dilutions of sera were mixed with vaccine virus, and after a 30 minute incubation at room temperature the serum/virus dilutions were added to erythrocyte suspensions in 96-well plates. After a 40 minute incubation at room temperature, wells were observed for agglutination. Endpoint titers were reported as the reciprocal of the highest dilution of serum that inhibited agglutination. The initial dilution was 1:4; agglutination observed in the first well would therefore be reported as a titer of <4, imputed for statistical purposes as a value of 2. The virus used in the assay was identical to that used in the vaccine, while the positive control serum used in the assays was generated in sheep using β-propiolactone–inactivated A/California/07/2009 virus as antigen. Specimens were assayed in duplicate, with the geometric mean of the 2 values being used in the analysis.
