# Supplementary material for: Safety and Immunogenicity Following Administration of a Live, Attenuated Monovalent 2009 H1N1 Influenza Vaccine to Children and Adults in Two Randomized Controlled Trials
Source: PLoS One. 2010 Oct 29;5(10):e13755. doi: 10.1371/journal.pone.0013755 (PMC2966412; doi:10.1371/journal.pone.0013755)
Supplement: Table S1 — Adverse Events Reported in Children ≤15 Days After Dose (Safety Population). (0.04 MB DOC) [file pone.0013755.s005.doc]

# Table S1. Adverse Events Reported in Children ≤15 Days After Dosing (Safety Population)

|  | Dose 1 | | Dose 2 | |
| --- | --- | --- | --- | --- |
| System Organ Class*  Event | H1N1 LAIV  (n=259) | Placebo  (n=65) | H1N1 LAIV  (n=255) | Placebo  (n=63) |
| Total number of events, n**^†^** | 67 | 16 | 46 | 10 |
| Total subjects reporting ≥1 event | 47 (18.1) | 11 (16.9) | 35 (13.7) | 9 (14.3) |
| Blood and lymphatic system disorders | NR | NR | 1 (0.4) | 0 (0.0) |
| Ear and labyrinth disorders | 3 (1.2) | 0 (0.0) | 1 (0.4) | 1 (1.6) |
| Eye disorders | 3 (1.2) | 0 (0.0) | 2 (0.8) | 0 (0.0) |
| Gastrointestinal disorders | 20 (7.7) | 5 (7.7) | 14(5.5) | 4 (6.3) |
| General disorders and administration site conditions | 4 (1.5) | 0 (0.0) | 2 (0.8) | 0 (0.0) |
| Immune system disorders | 1 (0.4) | 0 (0.0) | 1 (0.4) | 0 (0.0) |
| Infections and infestations | 6 (2.3) | 3 (4.6) | 14 (5.5) | 2 (3.2) |
| Injury, poisoning, and procedural complications | 10 (3.9) | 0 (0.0) | 3 (1.2) | 1 (1.6) |
| Musculoskeletal and connective tissue disorders | 2 (0.8) | 1 (1.5) | 1 (0.4) | 0 (0.0) |
| Nervous system disorders | 4 (1.5) | 0 (0.0) | NR | NR |
| Respiratory, thoracic and mediastinal disorders | 5 (1.9) | 1 (1.5) | NR | NR |
| Skin and subcutaneous tissue disorders | 2 (0.8) | 1 (1.5) | 2 (0.8) | 1 (1.6) |

NR=not reported

^*^MedDRA Version 12.0.

# ^†^Each subject may have had >1 recorded adverse event; however, each subject was counted only once per system organ class.
