# Supplementary material for: Safety and Immunogenicity Following Administration of a Live, Attenuated Monovalent 2009 H1N1 Influenza Vaccine to Children and Adults in Two Randomized Controlled Trials
Source: PLoS One. 2010 Oct 29;5(10):e13755. doi: 10.1371/journal.pone.0013755 (PMC2966412; doi:10.1371/journal.pone.0013755)
Supplement: Table S2 — Adverse Events Reported in Adults ≤15 Days After Dose (Safety Population). (0.04 MB DOC) [file pone.0013755.s006.doc]

# Table S2. Adverse Events Reported in Adults ≤15 Days After Dosing (Safety Population)

|  | Dose 1 | | Dose 2 | |
| --- | --- | --- | --- | --- |
| System Organ Class*  Event | H1N1 LAIV  (n = 240) | Placebo  (n = 60) | H1N1 LAIV  (n = 228) | Placebo  (n = 55) |
| Total number of events, n**^†^** | 53 | 14 | 23 | 4 |
| Total subjects reporting ≥1 event | 38 (15.8) | 10 (16.7) | 18 (7.9) | 4 (7.3) |
| Blood and lymphatic system disorders | 2 (0.8) | 0 (0.0) | 1 (0.4) | 0 (0.0) |
| Eye disorders | 3 (1.3) | 1 (1.7) | 1 (0.4) | 0 (0.0) |
| Gastrointestinal disorders | 12 (5.0) | 4 (6.7) | 5 (2.2) | 0 (0.0) |
| Immune system disorders | 1 (0.4) | 0 (0.0) | NR | NR |
| Infections and infestations | 3 (1.3) | 3 (5.0) | 4 (1.8) | 1 (1.8) |
| Injury, poisoning and procedural complications | 1 (0.4) | 0 (0.0) | NR | NR |
| Musculoskeletal and connective tissue disorders | 6 (2.5) | 0 (0.0) | NR | NR |
| Nervous system disorders | 2 (0.8) | 1 (1.7) | NR | NR |
| Psychiatric disorders | 0 (0.0) | 1 (1.7) | NR | NR |
| Respiratory, thoracic and mediastinal disorders | 14 (5.8) | 3 (5.0) | 8 (3.5) | 2 (3.6) |
| Skin and subcutaneous tissue disorders | 5 (2.1) | 0 (0.0) | 0 (0.0) | 1 (1.8) |
| Vascular disorders | 0 (0.0) | 1 (1.7) | NR | NR |

NR=not reported

^*^MedDRA Version 12.0.

# ^†^Each subject may have had >1 recorded adverse event; however, each subject was counted only once per system organ class.
