# Supplementary material for: Safety and Immunogenicity Following Administration of a Live, Attenuated Monovalent 2009 H1N1 Influenza Vaccine to Children and Adults in Two Randomized Controlled Trials
Source: PLoS One. 2010 Oct 29;5(10):e13755. doi: 10.1371/journal.pone.0013755 (PMC2966412; doi:10.1371/journal.pone.0013755)
Supplement: Protocol S2 — Adult Study MI-CP215. (1.74 MB PDF) [file pone.0013755.s008.pdf]

A Randomized, Double-blind, Placebo-controlled Study to Evaluate the Safety of MEDI3414 in Adults

**Sponsor Protocol Number:**	MI-CP215
**Application Number:**	BB-IND 14081
**Investigational Product:**	MEDI3414, Influenza Virus Vaccine, Monovalent, Type A (H1N1), Live, Cold-Adapted
**Sponsor:**	MedImmune<br>One MedImmune Way<br>Gaithersburg, MD 20878, USA
**Medical Monitor:**	Raburn Mallory, MD<br>Senior Director, Clinical Development<br>MedImmune<br>Phone: 301-398-5799; Fax: 301-398-8799
**Study Monitor:**	Quintiles CRO
**Protocol, Date:**	Original Protocol, 14May2009<br>Amendment 1, 22Jun2009

Sponsor Agreement:

Raburn Mallory, MD, Senior Director, Clinical Development, whose signature is on file at MedImmune, is authorized to sign the protocol on behalf of the sponsor.

Investigator Agreement:

I, the undersigned, have reviewed this protocol, and I agree to conduct this protocol in accordance with the International Conference on Harmonisation (ICH) guidelines on Good Clinical Practice (GCP), the ethical principles set forth in the Declaration of Helsinki, with the USA Code of Federal Regulations governing the protection of human subjects (21 CFR Part 50), Financial Disclosure by Clinical Investigators (21 CFR Part 54), Institutional Review Boards (21 CFR Part 56), and the obligations of clinical investigators (21 CFR Part 312) and other applicable local regulatory requirements.

The protocol may not be modified without written approval of the sponsor. All changes to the protocol must be submitted to the applicable regulatory authorities and Institutional Review Boards/Independent Ethics Committees (IRBs/IECs), and must be approved by the IRB/IEC prior to their implementation except when necessary to eliminate immediate hazards to the subjects or when the change(s), as deemed by the sponsor, involves only logistical or administrative changes. Documentation of IRB/IEC approval must be sent to the sponsor immediately upon receipt.

Signature: ________________________________ Date: ______________________

Investigator Name and Title: ____

(please print)

Investigator Affiliation, City, State/Province: ____________________________________

____

(please print)

Table of Contents

1 Introduction 12

1.1 Disease Background 12

1.2 Description of Investigational Vaccine 12

1.3 Nonclinical Experience with FluMist 13

1.4 Clinical Experience with FluMist 15

1.5 Rationale for Study 15

2 Study Objectives 16

2.1 Primary Objective 16

3 Study Design 16

3.1 Overview of Study Design 16

3.2 Estimated Study Duration 17

4 Study Procedures 17

4.1 Subject Participation and Identification 17

4.2 Subject Selection 18

4.2.1 Inclusion Criteria 18

4.2.2 Exclusion Criteria 19

4.3 Study Treatment Assignment 20

4.4 Blinding 21

4.5 Study Treatment 22

4.5.1 Investigational Product (Frozen Monovalent Vaccine or Placebo) 22

4.5.2 Treatment Regimens 23

4.5.3 Investigational Product Preparation and Administration 24

4.5.4 Concomitant Medications ... 24
4.5.5 Treatment Compliance ... 25
4.6 Subject Status ... 25
4.7 Study Completion ... 26
5 Assessment of Efficacy ... 26
5.1 Efficacy ... 26
5.2 Schedule of Study Procedures ... 26
5.2.1 Screening Visit (Day -14 to 1) ... 32
5.2.2 Dose 1 of Investigational Product (Day 1) ... 32
5.2.3 Follow-up Period Post Dose 1 of Investigational Product ... 33
5.2.3.1 Unscheduled Visit for Significant Febrile Illness Through Day 8 (Days 2-8) ... 33
5.2.3.2 Telephone Contacts During Days 2 to 16 ... 33
5.2.3.3 Post Dose 1 Immunigenicity Blood Draw Visit (Day 15 + 2) 33
5.2.4 Dose 2 of investigational Product (Day 29 + 2) ... 34
5.2.5 Follow-up Period Post Dose 2 of Investigational Product ... 35
5.2.5.1 Telephone Contacts During 14 Days Post Dose 2 ... 35
5.2.5.2 Post Dose 2 Immunogenicity Blood Draw Visit (Day 57 + 4) 35
5.2.5.3 Optional Vaccinations for Placebo Recipients to Receive Doses of Approved Product ... 35
5.2.5.4 Monthly Telephone Contacts (Days 61, 91, 121, 151, and 181) ... 36
5.2.5.5 Final Study Telephone Contact (Days 209-219) ... 36
5.3 Description of Study Procedures ... 36
5.3.1 Medical History and Physical Examination ... 36
5.3.2 Clinical Laboratory Tests ... 37
5.3.3 Immunogenicity Evaluation and Methods ... 37
5.3.4 Estimate of Volume of Blood ... 37

6 Assessment of Safety 38

6.1 Safety Parameters 38

6.1.1 Adverse Events 38

6.1.2 Serious Adverse Events 38

6.1.3 Solicited Symptoms 39

6.1.4 New Onset Chronic Disease 39

6.2 Assessment and Recording of Safety Parameters 40

6.2.1 Assessment of Severity 40

6.2.2 Assessment of Relationship 41

6.2.3 Recording of Adverse Events 42

6.2.4 Recording of Serious Adverse Events 42

6.2.5 Recording of Solicited Symptoms 43

6.2.6 Recording of New Onset Chronic Disease 43

6.3 Reporting Requirements for Safety Parameters 43

6.3.1 Study Reporting Period for Adverse Events 43

6.3.2 Study Reporting Period for Serious Adverse Events 44

6.3.2.1 Notification of Sponsor of Serious Adverse Events 44

6.3.2.2 Notification of Institutional Review Board or Independent Ethics Committee of Serious Adverse Events 45

6.3.3 Study Reporting Period for Solicited Symptoms 45

6.3.4 Study Reporting Period for New Onset of Chronic Disease 46

6.3.5 Other Events Requiring Immediate Reporting 46

6.3.5.1 Pregnancy and Overdose 46

6.3.5.2 Other Protocol-specific Events 46

6.4 Safety Management During the Study 47

6.4.1 Interruption or Permanent Discontinuation of Study Dosing in Individual Subjects 47

6.4.2 Study Stopping Criteria 48

6.4.3 Monitoring of Dose Administration 49

7 Statistical Considerations 49

7.1 General Considerations 49

7.2 Analysis Populations 49

7.3 Endpoints 50

7.3.1 Primary Endpoints 50

7.3.1.1 Primary Safety Endpoint 50

7.3.1.2 Primary Immunogenicity Endpoint 51

7.3.2 Secondary Endpoints 51

7.3.2.1 Secondary Safety Endpoints 51

7.3.2.2 Secondary Immunogenicity Endpoints 52

7.4 Planned Data Submissions 52

7.5 Sample Size and Power Calculations 53

7.6 Multiplicity Considerations 55

8 Direct Access to Source Data and Documents 55

9 Quality Control and Quality Assurance 56

9.1 Data Collection 56

9.2 Study Monitoring 56

9.3 Audit and Inspection of the Study 57

10 Ethics 57

10.1 Regulatory Considerations 57

10.2 Institutional Review Board or Independent Ethics Committee 58

10.3 Informed Consent 58

11 Data Handling and Record Keeping 60

12 Financing and Insurance 60

13 Publication Policy 60

14 References 61

15 Summary Protocol Amendments and Administrative Changes to the Protocol 62

List of In-text Tables

Table 5.2-1 Schedule of Evaluations 28

Table 7.5-1 Summary of Solicited Events (Days 0-7) in Study AV009 53

Table 7.5-2 Power to Rule Out a 10 Percentage Point Increase of Fever Rate in Vaccine Recipients 54

Table 7.5-3 Power for Secondary Endpoints 55

List of Appendices

Appendix 1 FluMist Full Prescribing Information 63

Appendix 2 Intranasal Sprayer Administration Instructions 82

List of Abbreviations

Abbreviation	Definition
AE	adverse event
ca	cold adapted
att	attenuated
CDC	Centers for Disease Control and Prevention
CI	confidence interval
CRF	case report form
CTM	clinical trial material
EU	European Union
FDA	Food and Drug Administration
FFU	fluorescent focus units
GCP	Good Clinical Practice
GMT	geometric mean titer
HAI	hemagglutination inhibition
HIPAA	Health Insurance Portability and Accountability Act
ICH	International Conference on Harmonisation
IEC	Independent Ethics Committee
IRB	Institutional Review Board
IRE	immediately reportable event
ITT	intent-to-treat
IVRS	Interactive Voice Response System
MedDRA	Medical Dictionary for Regulatory Activities
NA	not applicable
NOCD	new onset chronic disease
SAE	serious adverse event
SID	subject identification
ts	temperature sensitive
WHO	World Health Organization

Study Abstract

TITLE

A Randomized, Double-blind, Placebo-controlled Study to Evaluate the Safety of MEDI3414 in Adults

OBJECTIVES

The primary objective of this study is to assess the safety and descriptive immunogenicity of a monovalent influenza virus vaccine containing a new 6:2 influenza virus reassortant in healthy adults.

STUDY DESIGN

This prospective, randomized, double-blind, placebo-controlled product release study will enroll approximately 300 adults 18-49 years of age. Eligible subjects will be randomly assigned in a 4:1 ratio to receive two doses of active monovalent vaccine or placebo by intranasal spray; the doses will be administered approximately 28 days apart, on Days 1 and 29. This study will be conducted at multiple sites in the United States. Randomization will be stratified by site.

Subjects will be screened for the study within 14 days prior to randomization. On Day 1, prior to dosing, a baseline blood sample for assessment of immune response will be obtained. Subjects will receive the first dose of investigational product (active or placebo) at the site on Day 1. Subjects will also be given memory aid worksheets to record solicited symptoms during the first 14 days after the Day 1 dose. In the event that a subject experiences a febrile illness during the first 7 days after the Day 1 dose, the subject should return to the site for evaluation (ie, an unscheduled visit). For each treatment group, subjects will be randomized at a 1:1 ratio to have their post Dose 1 immunogenicity blood draw occur on either Day 15 or Day 29. All subjects will return to the site for a visit on Day 29. Subjects will receive the second dose of investigational product (active or placebo) on Day 29 and will be given memory aid worksheets to record solicited symptoms during the 14-day period after the second dose. All subjects will return to the site on Day 57 (or approximately 28 days post Dose 2) for collection of a blood sample to assess immune response post Dose 2.

Telephone contacts will be made by site personnel to the subject at various times during the study to assess safety. Following Dose 1, telephone contacts will be made every other day during Days 2-8, on Day 9, and every other day during Days 10-16. Following Dose 2, telephone contact will be made every other day during Days 30-44, and once during each of the following intervals: Days 61-68, Days 91-98, Days 121-128, Days 151-158, Days 181-188, and Days 209-219. The final study contact will be the Day 209-219 telephone contact, approximately 180 days after the second dose of investigational product.

Safety evaluations will consist of the collection of solicited symptoms, adverse events (AEs), and concomitant medication use during the 14-day period (ie, Days 1 to 15 and approximately Days 29 to 43) after receipt of each dose of investigational product. Serious adverse events (SAEs) and new onset of chronic disease (NOCD) will be collected through 180 days after receipt of the final dose of investigational product.

Five data submissions to the United States Food and Drug Administration (FDA) are planned in the study (see Planned Analyses section).

After the strain-change supplement has been approved by FDA, subjects who were randomized to placebo will be offered the option of receiving dosing with approved product. For subjects who agree, two doses of the approved product will be administered by site personnel approximately 28 days apart. The approved formulation provided will not be the exact commercial formulation; it will be the active monovalent vaccine administered in this study. The exact timing of this will depend on approval by the FDA. The first optional dose of approved product may not be administered until after the Day 57 blood sample for hemagglutination inhibition (HAI) testing has been collected (see Section 5.2.5.2).

The total duration of subject participation in this study, including a screening period of up to 14 days, is anticipated to be approximately 7 to 8 months.

Study Abstract

**SUBJECT POPULATION**<br>The subjects in this study will be healthy adults 18 to 49 years of age (not yet reached their 50th birthday at time of randomization).
**TREATMENT**<br>Eligible subjects will be randomly assigned in a 4:1 ratio to receive two doses of investigational product (either frozen monovalent vaccine or placebo) by intranasal spray on Days 1 and 29.
**ASSESSMENT OF ENDPOINTS**<br>Primary safety endpoint:<br>The primary safety endpoint of this study is fever (Days 1-8), defined as an oral temperature ≥ 101°F (38.3°C). The rate of subjects with fever between the two treatment groups will be compared based on the upper limit of the two-sided 95% exact confidence intervals (CIs) for the rate difference (Vaccine minus Placebo). A two-sided 95% CI will be constructed using the exact method based on the score statistic proposed by Chan and Zhang (Chan and Zhang 1999). The upper limit of the two-sided 95% CI of the rate difference will be evaluated against the prespecified equivalence criterion of 10%, which corresponds to the following hypotheses:<br>H0 (null): Rate Difference ≥ 10%<br>HA(alternative): Rate Difference < 10%<br>Primary immunogenicity endpoint:<br>The primary immunogenicity endpoint of the study is the proportion of subjects who experience a post dose seroresponse against the H1N1 strain in baseline seronegative subjects and in all subjects regardless of baseline serostatus, by dose number.<br>Secondary safety endpoints of the study include other solicited symptoms, antipyretic and analgesic use, and AEs that occur within 7 days after each vaccination with investigational product (Days 1-8 and Days 29-36). Additional secondary safety endpoints include all solicited symptoms, antipyretic and analgesic use, AEs and SAEs that occur within 14 days after each vaccination with investigational product (ie, Days 1-15 and Days 29-43), and SAEs and NOCDs that occur within 28 days after each vaccination with investigational product (ie, Days 1-29 and Days 29-57) and through study completion (Day 209).<br>Secondary immunogenicity endpoints of the study include the post dose strain-specific serum HAI antibody geometric mean titers (GMTs) by baseline serostatus and the proportion of subjects who achieve a post dose strain-specific HAI titer > 32 in baseline seronegative subjects and in all subjects regardless of baseline serostatus, by dose number.

Study Abstract

PLANNED ANALYSES

Five data submissions are planned in the study. The primary assessment of safety will be performed at Day 8. To summarize the primary safety data (Days 1-8) and report the strain-change results to the FDA, the study will be unblinded to MedImmune personnel at the Day 8 assessment. The assessment of immunogenicity will be performed at Day 15 (14 days post Dose 1), Day 29 (28 days post Dose 1), and Day 57 (28 days post Dose 2). The Day 29 analysis will also include all safety data available through Day 29 (28 days post Dose 1), as well as preliminary Dose 2 safety data (7 days post Dose 2). The Day 57 analysis will also include all safety data available through approximately Day 43 (14 days post Dose 2). The final study analysis will be performed at the conclusion of the study for all data through Day 209.

All site personnel and subjects will remain blinded to treatment assignments through Day 57 of the last subject randomized and dosed. Once the strain-change supplement has been approved by the FDA, study site personnel and subjects will be unblinded, so that subjects initially randomized to receive placebo can be offered two optional doses of the approved product, to be administered at the study site approximately 28 days apart.

SAMPLE SIZE AND POWER CALCULATIONS

The sample size and power calculations are based on the safety endpoints. Based on the solicited event rates following FluMist and placebo vaccination in healthy adults 18-64 years of age in Study AV009, the currently proposed study with 300 evaluable subjects (240 vaccine and 60 placebo recipients) will provide at least >99.9% power to rule out a rate increase of 10 percentage points, assuming the true difference between the treatment groups is zero and the true fever rate is ≤ 2%. Power is also high if the true difference is slightly greater than zero and the true fever rate is ≤ 3%. For example, if the true fever rates are 3% and 1% in active and placebo recipients, respectively, the power will be 99.6%. For other solicited symptoms, the currently proposed study design provides at least 87% power to rule out a rate difference of 20 percentage points, assuming the true difference between the two treatment groups is zero. Power is lower if the true difference is greater than zero.

1 Introduction

1.1 Disease Background

In the spring of 2009, an outbreak of respiratory illnesses was first noted in 3 clusters in Mexico, which were eventually identified as being related to influenza A (H1N1) (World Health Organization [WHO], April 2009). As of 18Jun2009, more than 70 countries have officially reported 39,620 cases of influenza A (H1N1) infection including 167 deaths (WHO, 18Jun2009). Cases have occurred in both the Northern and Southern Hemispheres. This outbreak of influenza A (H1N1) infection appears to involve sustained human-to-human transmission, as suggested by the large numbers of patients with respiratory illnesses identified within a short period of time at various locations around the world. In response to this sustained human-to-human transmission, the WHO raised its pandemic alert level to Phase 6 on 11Jun2009. The outbreak is due to a new strain of influenza A virus subtype H1N1 that derives by reassortment from one strain of human influenza virus, one strain of avian influenza virus, and two separate strains of swine influenza virus.

As of 12Jun2009, 17,855 confirmed or probable cases of influenza A (H1N1) infection have also been reported from all 50 states and the District of Columbia in the United States (Centers for Disease Control and Prevention [CDC], 12Jun2009). The majority of cases in the United States have been mild, although more than 100 patients have required hospitalization and 44 patients have died.

During the 2009 outbreak, rapidly progressive pneumonia, respiratory failure, and acute respiratory distress syndrome have been reported in some cases in Mexico (CDC, 27Apr2009). In the United States, most described cases to date have been mild as of 18Jun2009. The signs and symptoms of influenza caused by influenza A (H1N1) virus are similar to those of seasonal influenza (CDC, 01Jun2009). Typical clinical manifestations include fever, headache, cough, sore throat, myalgias, chills, and fatigue. Diarrhea and vomiting may also occur. The range of complications of infection with the current strain of influenza A (H1N1) virus is not yet known.

1.2 Description of Investigational Vaccine

MEDI3414 [Influenza A (H1N1) vaccine, live attenuated, intranasal] is an intranasally administered, live attenuated monovalent influenza vaccine delivered by the Becton

Dickinson (BD) Accuspray™ device that is intended for the active immunization of individuals 2 to 49 years of age against influenza disease caused by the influenza virus A (H1N1) subtype contained in the vaccine. It is formulated to contain $10^{7.0 \pm 0.5}$ Fluorescent Focus Units (FFU) per dose of the A/California/7/2009 strain of the live, attenuated influenza virus reassortant that was propagated in chicken eggs. MEDI3414 contains no preservatives and no adjuvants. The reassortant influenza virus strain in MEDI3414 is cold-adapted (*ca*) (ie, it replicates efficiently at 25°C, a temperature that is restrictive for replication of many wild-type influenza viruses), temperature sensitive (*ts*) (ie, it is restricted in replication at 39°C, temperatures at which many wild-type influenza viruses grow efficiently), and attenuated (*att*) (ie, it does not produce classic influenza-like illness in the ferret model of human influenza infection) (Jin et al, 2003; Hoffmann et al, 2005; Murphy and Coelingh, 2002). The cumulative effect of the *ca*, *ts*, and *att* phenotypes is that replication of the attenuated vaccine virus is restricted to the nose and pharynx, where the vaccine is thought to induce both localized and systemic protective immunity (ie, humoral, mucosal, and cellular) similar to that induced after infection with wild-type virus.

MEDI3414 is a monovalent strain formulated with sucrose-phosphate buffer in a total volume of 0.5 mL in the BD Accuspray™ device. The MEDI3414 clinical trial material (CTM) is similar to the CTM used in the 2009-2010 annual safety study for FluMist® (MI-MA205, submitted to BB-IND 9204), with the exception that the strain used in that study was B/Brisbane/60/2008, the new 6:2 reassortant strain to be included in the 2009-2010 commercial FluMist® formulation, rather than the A/California/7/2009 strain used for the current CTM.

1.3 Nonclinical Experience with FluMist

A number of nonclinical studies on both the former frozen and current refrigerated formulations of FluMist have been conducted in ferrets, as the ferret has been proven to be a good model for studying influenza.

Primary pharmacodynamic studies in ferrets have been conducted to evaluate the replication and immunogenicity of live, attenuated FluMist strains. Results of ferret replication, immunogenicity, and challenge studies demonstrated that vaccination with both frozen and refrigerated formulations of FluMist protected animals following challenge with wild-type virus; vaccine-induced immunity in these animals substantially decreased replication of the wild-type virus in the lungs as well as the upper airways.

Toxicology studies performed with FluMist include a repeat dose toxicology study in ferrets, two reproductive toxicology studies in rats and ferrets, and two ocular toxicology studies (Draize tests) in a rabbit model to evaluate the potential effects of FluMist if inadvertently sprayed into the eye.

A single and repeated dose toxicology study was conducted in ferrets to investigate the potential adverse effects of refrigerated FluMist given one or three times to ferrets over a 15-week period. The regimen consisted of up to 3 human doses of FluMist administered intranasally at Weeks 0, 4 and 14. This dosing regimen is in compliance with ICH M3 which recommends a toxicity study duration of 3 months with a product dosing duration of up to 1 month. No clinical indications of toxicity were manifest during the course of the study from any of the parameters evaluated. No test material-related toxicity was identified in the major organs examined histopathologically except for increased incidence of acute, multifocal, suppurative inflammation of the nasal turbinates and lymph node hyperplasia in animals at interim necropsy. These findings were most likely due to inoculation 3 days prior to necropsy and the antigenic responses of the animals to the inoculum. Overall, the vaccine was well tolerated.

Studies were conducted to evaluate reproductive toxicology in two different animal models (rat and ferret). Results of the study conducted with rats indicated that exposure to FluMist once prior to mating and once during pregnancy did not produce any maternal toxicity or affect the reproductive capacity of the dam. These exposures did not produce embryo fetal toxicity in near-term fetuses or pups evaluated for 21 days postpartum.

The second study involved intranasal administration of FluMist to pregnant ferrets at 4 different time points during gestation. No vaccine treatment-related effects were observed with respect to maternal mortality, clinical observations, or body weight during gestation, nor were there any treatment-related effects observed in fetal parameters or in maternal macroscopic pathology that could be attributed to the intranasal instillation of FluMist.

The potential for ocular toxicity resulting from the inadvertent instillation of FluMist into the eye was evaluated in two ocular toxicity studies in rabbits using a standard Draize test. Neither study elicited results consistent with ocular toxicity.

The results of the nonclinical studies performed on both frozen and refrigerated formulations of FluMist collectively demonstrate that the vaccine has a favorable safety and tolerability profile.

1.4 Clinical Experience with FluMist

In 57 clinical studies, more than 120,000 subjects from 6 weeks to > 90 years of age have been administered MedImmune's egg-produced vaccine, FluMist. These studies were designed to evaluate the safety, efficacy, and immunogenicity of the vaccine among various study populations, including > 90,000 children and adolescents 6 weeks to 17 years of age and > 30,000 adults 18 to > 90 years of age. In these studies, FluMist was found to be generally safe and well tolerated. In healthy adults 18-64 years of age, FluMist recipients reported higher rates of runny nose (44%) and sore throat (26%) than placebo recipients (27% and 17%, respectively), with a median event duration of 1 day regardless of treatment received. Other symptoms, including cough, headache, chills, muscle aches, and tiredness/weakness occurred less often and at similar rates between the treatment groups. Low-grade fever (temperature > 100ºF) occurred at a similarly low rate for FluMist and placebo recipients (1.3% and 1.5%).

In addition to the clinical study experience, over 70,000 doses of FluMist have been administered in two post-marketing studies, and more than 17 million doses of FluMist have been distributed commercially in the USA from initial licensure in 2003 to the end of the 2008-2009 influenza season. FluMist was initially approved for use in healthy individuals 5 to 49 years of age; however, the age indication was expanded to include children 24-59 months of age prior to the 2007-2008 season. To date, no unexpected serious risks have been identified for FluMist when used according to approved indications.

See the current FluMist full prescribing information in Appendix 1 for additional details.

1.5 Rationale for Study

The purpose of this study is evaluate the safety and immunogenicity of an investigational monovalent vaccine that is being developed for the active immunization of individuals 2-49 years of age against influenza disease caused by influenza virus subtype A.

The design of this clinical study will ensure that the new vaccine virus has sufficient attenuation in humans, as demonstrated by the low incidence of fever ≥ 101°F (39.3°C). In previous clinical studies, the rate of fever in adults following administration of FluMist has been low and similar in both vaccinees and placebo recipients. For example, in Study AV009 conducted in 4,561 healthy adults 18-64 years of age (3,041 FluMist recipients and 1,520 placebo recipients), fever ≥ 101°F occurred in 0.57% and 0.60% of FluMist and placebo

recipients, respectively. Therefore, in a placebo-controlled study in healthy adults to evaluate new strains, vaccine recipients would be expected to develop temperature elevations at rates similar to those of placebo recipients following vaccination.

2 Study Objectives

2.1 Primary Objective

The primary objective of this study is to assess the safety and descriptive immunogenicity of a monovalent influenza virus vaccine of a new 6:2 influenza virus reassortant in healthy adults.

3 Study Design

3.1 Overview of Study Design

This prospective, randomized, double-blind, placebo-controlled product release study will enroll approximately 300 healthy adults 18 to 49 years of age. Eligible subjects will be randomly assigned in a 4:1 ratio to receive two doses of active monovalent vaccine or placebo by intranasal spray; the doses will be administered approximately 28 days apart, on Days 1 and 29. This study will be conducted at multiple sites in the United States. Randomization will be stratified by site.

Subjects will be screened for the study within 14 days prior to randomization. On Day 1, prior to dosing, a baseline blood sample for assessment of immune response will be obtained. Subjects will receive the first dose of investigational product (active or placebo) at the site on Day 1. Subjects will also be given memory aid worksheets to record solicited symptoms during the first 14 days after the Day 1 dose. In the event that a subject experiences a febrile illness during the first 7 days after the Day 1 dose, the subject should return to the site for evaluation (ie, an unscheduled visit). For each treatment group, subjects will be randomized at a 1:1 ratio to have their post Dose 1 immunogenicity blood draw occur on either Day 15 or Day 29. Subjects will receive the second dose of investigational product (active or placebo) on Day 29 and will be given memory aid worksheets to record solicited symptoms during the 14-day period after the second dose. All subjects will return to the site on Day 57 (or approximately 28 days after dose 2) for collection of a blood sample to assess immune response.

Telephone contacts will be made by site personnel to the subject at various times during the study to assess safety. Telephone contacts will be made every other day during Days 2-8, on Day 9, every other day during Days 10-16, and every other day during Days 30-44. One telephone contact will be made during each of the following intervals: Days 61-68, Days 91-98, Days 121-128, Days 151-158, Days 181-188, and Days 209-219. The final study contact will be the Day 209-219 telephone contact, approximately 180 days after the second dose of investigational product.

Safety evaluations will consist of the collection of solicited symptoms, adverse events (AEs) and concomitant medication use during the 14-day period (ie Days 1 to 15 and approximately Days 29 to 43) after receipt of each dose of investigational product. Serious adverse events (SAEs) and new onset of chronic disease (NOCD) will be collected through 180 days after receipt of the final dose of investigational product (ie, approximately Day 209-211).

All site personnel and subjects will remain blinded to treatment assignments through Day 57, ie 28 days after the last subject has received Dose 2 of the investigational product. Once the strain-change supplement has been approved by the FDA, study site personnel and subjects will be unblinded, so that subjects initially randomized to receive placebo can be offered 2 optional doses of the approved product, to be administered at the study site approximately 28 days apart.

The endpoints to be measured in this study are described in Section 7.3.

3.2 Estimated Study Duration

The total duration of subject participation in this study, including a screening period of up to 14 days, is anticipated to be approximately 7 to 8 months.

4 Study Procedures

4.1 Subject Participation and Identification

Study participation begins once written informed consent is obtained (see Section 10.3 for details). Once informed consent is obtained, a subject identification (SID) number will be assigned using an interactive voice response system (IVRS), and the screening evaluations may begin to assess study eligibility (inclusion/exclusion) criteria. The SID number will be

used to identify the subject during the screening process and throughout study participation, if applicable.

A master log of all consented subjects will be maintained at the site and will document all screening failures (ie, subjects who are consented but do not meet study eligibility criteria and/or are not randomized), including the reason(s) for screening failure.

4.2 Subject Selection

The subjects in this study will be healthy adults 18 to 49 years of age (not yet reached their 50th birthday at time of randomization).

The investigator (physician) or qualified designee will discuss the study with a subject/the legal representative of a subject who is considered a potential candidate for the study and provide the subject/legal representative with the study-specific informed consent form approved by the IRB/IEC. The investigator or designee will address any questions and/or concerns that the subject/legal representative may have and, if there is continued interest, will secure written informed consent for participation in the study. Written informed consent and any locally required authorization (eg, Health Insurance Portability and Accountability Act [HIPAA] authorization in the USA, European Union [EU] Data Privacy Directive authorization in the EU, and written informed assent) will be obtained prior to conducting any protocol-related procedures, including screening evaluations or medication washouts. See Section 10.3 for additional details concerning informed consent.

4.2.1 Inclusion Criteria

Subjects must meet *all* of the following criteria:

1) Male or female, 18 to 49 years of age (not yet reached their 50th birthday) at the time of randomization
2) Healthy by medical history and physical exam
3) Written informed consent and any locally required authorization (eg, HIPAA in the USA, EU Data Privacy Directive in the EU) obtained from the subject/legal representative prior to performing any protocol-related procedures, including screening evaluations
4) Females of childbearing potential, (ie, unless surgically sterile [eg, bilateral tubal ligation, bilateral oophorectomy, or hysterectomy], has sterile male partner, is at least 1 year post menopause, or practices abstinence) must use an effective method of

avoiding pregnancy (including oral, transdermal, or implanted contraceptives, intrauterine device, female condom with spermicide, diaphragm with spermicide, cervical cap, or use of a condom with spermicide by the sexual partner) for 30 days prior to the first dose of investigational product, and must agree to continue using such precautions for 60 days after the second dose of investigational product. In addition, the subject must also have a negative urine or blood pregnancy test at screening and, if screening and Day 1 do not occur on the same day, on the day of vaccination prior to randomization.

5) Males, unless not sexually active, must use an effective method of birth control with a female partner and must agree to continue using such contraceptive precautions for at least 30 days after the second dose of investigational product (from Day 1 through Day 59 of the study)
6) Subject is available by telephone
7) Subject is able to understand and comply with the requirements of the protocol, as judged by the investigator
8) Ability to complete follow-up period of 180 days after Dose 2 as required by the protocol

4.2.2 Exclusion Criteria

Any of the following would exclude the subject from participation in the study:

1) History of hypersensitivity to any component of the investigational product including egg or egg protein, gelatin or arginine, or serious, life threatening, or severe reactions to previous influenza vaccinations
2) History of hypersensitivity to gentamicin
3) Any condition for which the **inactivated** influenza vaccine is indicated, including chronic disorders of the pulmonary or cardiovascular systems (eg, asthma), chronic metabolic diseases (eg, diabetes mellitus), renal dysfunction, or hemoglobinopathies that required regular medical follow-up or hospitalization during the preceding year
4) Acute febrile (> 100.0°F oral or equivalent) and/or clinically significant respiratory illness (eg, cough or sore throat) within 14 days prior to randomization
5) History of asthma
6) Any known immunosuppressive condition or immune deficiency disease, including HIV infection, or ongoing immunosuppressive therapy
7) History of Guillain-Barré syndrome
8) A household contact who is severely immunocompromised (eg, hematopoietic stem cell transplant recipient, during those periods in which the immunocompromised individual requires care in a protective environment); subject should additionally

avoid close contact with severely immunocompromised individuals for at least 21 days after receipt of investigational product

9) Receipt of any investigational agent within 30 days prior to randomization, or expected receipt through 30 days after the second dose of investigational product (use of licensed agents for indications not listed in the package insert is permitted)

10) Expected receipt of antipyretic or analgesic medication on a daily or every other day basis from randomization through 14 days after receipt of each dose of investigational product

11) Administration of intranasal medications within 14 days prior to randomization, or expected receipt through 14 days after administration of each dose of investigational product

12) Receipt of any nonstudy vaccine within 30 days before or after Dose 1 or expected receipt of any nonstudy vaccine within 30 days before or after Dose 2.

13) Known or suspected mitochondrial encephalomyopathy

14) Subject is pregnant or a nursing mother

15) Any condition (eg, chronic cough, allergic rhinitis) that, in the opinion of the investigator, would interfere with evaluation of the investigational product or interpretation of subject safety or study results

16) Subject or immediate family member of subject is an employee of the clinical study site or is otherwise in involved with the conduct of the study

4.3 Study Treatment Assignment

An IVRS will be used for randomization to a treatment arm and assignment of blinded investigational product kit numbers. A subject is considered randomized into the study when the investigator notifies the IVRS that the subject meets eligibility criteria and the IVRS provides the assignment of a blinded investigational product kit number to the subject.

Subjects will be randomized at a 4:1 ratio to receive either monovalent vaccine or placebo. For each treatment group, subjects will be further randomized at a 1:1 ratio to have their post Dose 1 immunogenicity blood draw occur on either Day 15 or Day 29. The randomization will be stratified by site.

The procedure for using the IVRS for both randomization and assignment of the first dose investigational product kit number on Day 1 as well as assignment of the second dose investigational product kit number on Day 29 is as follows:

- The investigator or designee contacts the IVRS and provides the SID number and subject's baseline characteristic(s) used when the original SID number was assigned (see Section 4.1), to verify that it is the same subject
- The IVRS assigns a treatment arm and investigational product kit number(s) to the subject
- A confirmatory communication with this information is sent to the investigator/designee who dispenses the investigational product to the subject per the communication and records the appropriate information in the subject's medical records and investigational product accountability log

Each dose of investigational product (monovalent vaccine or placebo) must be administered within 1 hour of thawing of the investigational product. If there is a delay in the administration of investigational product such that it will not be administered within the specified timeframe, the study monitor must be notified immediately.

4.4 Blinding

This is a double-blind study through the period of analysis for the study's primary safety endpoint (ie, through the Day 8 safety analysis). Active monovalent vaccine and placebo in this study are identically labeled and indistinguishable in appearance; therefore, subjects/legal representatives and study site personnel, including the investigators, study nurses, coordinators, and investigator's or site's designated investigational product manager, will be blinded to treatment assignment. An interim assessment of safety will be performed at Day 8. To summarize the primary safety phase data (Days 1-8) and report the primary safety end-point results to FDA, the study will be unblinded to MedImmune personnel at the Day 8 assessment.

All MedImmune personnel or designees who are directly involved with the conduct of the study will remain blinded to treatment assignments until the database is locked and unblinded for the Day 8 safety analysis. Because the immunogenicity endpoints are serologic, laboratory staff will remain blinded to all subject treatment assignments until after completion of immunogenicity analyses. Study site personnel and subjects will remain blinded through collection of Day 57 data. Once the strain-change supplement has been approved by the FDA, study site personnel and subjects will be unblinded, so that subjects initially randomized to receive placebo can be offered two optional doses of the approved product to be administered at the study site (see Section 5.2.5.3).

The vendor for packaging and labeling of the clinical supplies, designated IVRS personnel, designated MedImmune Clinical Research Pharmacy Service (CRPS) personnel, designated persons in Clinical Operations, are the only individuals who will have access to information that may identify a subject's treatment allocation prior to the unblinding for the Day 8 analysis. These individuals must not reveal randomization or treatment information to anyone or participate in or be associated with the evaluation of study subjects. In the event that the treatment allocation for a subject becomes known to the investigator or other study staff or needs to be known to treat an individual subject for an AE prior to the specified time for unblinding of sites to this information, the sponsor must be notified *immediately* by the investigator.

In an emergency, the investigator will have the ability to become unblinded. Any unblinding due to an emergent safety issue will be performed according to the IVRS manual.

4.5 Study Treatment

4.5.1 Investigational Product (Frozen Monovalent Vaccine or Placebo)

Investigational product will be distributed to clinical sites using designated distribution centers. The sponsor will provide the investigator(s) with adequate quantities of investigational product.

Monovalent Vaccine: Monovalent vaccine is supplied in intranasal sprayers containing a total volume of 0.5 mL of sucrose-phosphate buffer, egg allantoic fluid, and approximately 10^7 FFU of a monovalent influenza virus type A/California/7/2009.

Placebo: Placebo is supplied in intranasal sprayers containing 0.5 mL of sucrose-phosphate buffer.

Investigational product (active monovalent vaccine or placebo) will be supplied to the site in devices with identical appearances in coded kits. Each kit has a unique number that is printed on all labels within the kit (ie, the outer carton label and the label of each device within the carton). Detailed instructions are in the Investigational Product Manual supplied by MedImmune. The sprayer labels affixed to the investigational product will not identify the treatment group allocation.

On request by MedImmune or its designee, investigational product will be packaged and shipped frozen (on dry ice) directly to the clinical study site by express courier. Receiving departments should be notified that rapid handling of the shipment is required. Upon receipt at each study site, frozen investigational product should be immediately transferred to a -20°C (5°F) freezer. Freezer temperature variations up to ± 5°C are permitted. The manufacturer's instructions for shipment and storage will be followed at all times. It is the responsibility of the investigator to maintain a daily temperature log for the freezer (refer to the Investigational Product Manual for details). The investigator will be provided with temperature monitors that record minimum/maximum temperatures, unless temperature monitors are already in place.

Administration instructions are included in Section 4.5.3 and Appendix 2. Additional details regarding investigational product supplies, dose preparation, and accountability will be provided in the Investigational Product Manual supplied to the sites. In the event that an assigned study sprayer is broken or damaged, or if there is any concern about the integrity of the closed system of the sprayer, a call to the IVRS will allow for study site personnel to obtain the appropriate replacement sprayer number when necessary. The replacement sprayer will contain the same study material as the originally assigned sprayer. Refer to the Investigational Product Manual for more detailed information regarding investigational product replacement procedures.

The investigator's or site's designated investigational product manager is required to maintain accurate investigational product accountability records. Upon completion of the study, copies of investigational product accountability records will be returned to the sponsor. All unused investigational product will be returned to a MedImmune-authorized depot or disposed of upon authorization by MedImmune (refer to the Investigational Product Manual or other written instructions provided by MedImmune or its designee for contact information and specific shipping instructions).

4.5.2 Treatment Regimens

Eligible subjects will be randomly assigned in a 4:1 ratio to receive 2 doses of active monovalent vaccine (240 subjects) or placebo (60 subjects) by intranasal spray; the doses will be administered approximately 28 days apart on Days 1 and 29.

4.5.3 Investigational Product Preparation and Administration

- Investigational product should be brought to room temperature by holding the sprayer in the palm of the hand and supporting the plunger rod with the thumb (see Appendix 2). The vaccine should be administered within 1 hour of thawing.
- At room temperature, investigational product is a colorless to pale yellow liquid, and clear to slightly cloudy. Some proteinaceous particles may be present but they do not affect the use of the product.
- A single administration comprises intranasal delivery of approximately 0.5 mL total volume (0.25 mL into each nostril). Each sprayer has a divider that allows delivery of approximately half the contents of the sprayer into one nostril. Removal of the divider allows delivery of the remaining volume into the other nostril.
- The individual administering the vaccine should depress the plunger rod as rapidly as possible to generate a fine mist. Half of the contents of each sprayer will be sprayed as a fine mist into each nostril while the subject is in an upright position.
- After administration, used study sprayers must be placed immediately after use into locked containers or sealed bags.
- After vaccination, all subjects will be observed for a minimum of 15 minutes by the study staff. Emergency management supplies (eg, AMBU bag, adrenaline [epinephrine], antihistamine) will be made available for the initial treatment of an allergic reaction if needed. Local reactions or systemic events must be recorded.

The day of receipt of the first dose of investigational product is considered Day 1.

4.5.4 Concomitant Medications

Use of concomitant medications from investigational product administration through 14 days after each vaccination with investigational product is discouraged. However, subjects may receive medications as supportive care or to treat AEs as deemed necessary by the investigator or the subject's physician. Use of anti-pyretic or analgesic medications for all symptoms other than temperatures ≥ 101°F (≥ 38.3°C) oral (or equivalent) is discouraged during the 14 day period after each dose of investigational product. All medications taken during the 14-day post vaccination follow-up period, the indications, the start and stop dates of the medications, and the route of administration will be recorded in the subject's record.

All concomitant medications given to the subject during the 14-day post vaccination follow-up period after both doses of investigational product will be recorded on the source document.

The sponsor must be notified if a subject receives any of these during the study.

1) Any investigational agent or vaccine through 30 days post each dose of investigational product administration
2) Antipyretic or analgesic medication through 14 days post each dose of investigational product administration
3) Any intranasal medication through 14 days post each dose of investigational product administration
4) Receipt of antiviral therapy or antiviral agents within 48 hours prior to investigational product administration or expected receipt of antiviral therapy or antiviral agents through 14 days after receipt of each dose of investigational product

4.5.5 Treatment Compliance

Investigational product is administered by study site personnel, who will monitor compliance.

4.6 Subject Status

Subject Completion

An individual subject will be considered to have completed the primary safety assessment period of the study if the subject provides safety data through Day 8. An individual subject will be considered to have completed the study if the subject experienced a successful final study contact at least 180 days after receipt of the second (Day 29) dose of investigational product for the collection of any serious adverse events (SAEs) or new onset chronic disease (NOCD).

Subjects will be considered not to have completed the study if one of the following conditions applies:

- Withdrawal of consent: If consent for follow-up is withdrawn, the subject will not receive any further investigational product or further study observation
- Lost to follow-up: Subjects will be considered lost-to-follow-up only if no contact has been established by the time the study is completed such that there is insufficient information to determine the subject's status at the final scheduled study contact approximately 180 days after the Day 29 dose (ie, at approximately Day 209-211).

Note: Subjects refusing to return to the site or to continue participation in the study should be documented as "withdrawal of consent" rather than "lost to follow-up." Investigators should

document attempts to re-establish contact with missing subjects throughout the study period. If contact with a missing subject is re-established, the subject should not be considered lost-to-follow-up and any evaluations should resume according to the protocol.

Permanent Discontinuation of Investigational Product

Subjects who are permanently discontinued from further receipt of investigational product, regardless of the reason (withdrawal of consent, due to an AE, other), will be identified as having permanently discontinued treatment (Section 6.4.1).

Permanent discontinuation of investigational product does not apply to subjects who do not receive more than 2 doses of investigational product, ie, refusal by a subject who received placebo on Day 1 to receive the optional doses of active monovalent vaccine after approval by FDA does not constitute permanent discontinuation of investigational product.

4.7 Study Completion

Overall study completion is defined as the date of the last protocol-specified visit/assessment (including telephone contact at Day 209) for the last subject in the study. All materials or supplies provided by the sponsor will be returned to the sponsor or designee upon study completion, as directed by the site monitor. The investigator will notify the IRB/IEC when the study has been completed.

5 Assessment of Efficacy

5.1 Efficacy

Investigational product efficacy will not be assessed in this study.

5.2 Schedule of Study Procedures

All subjects who are assigned an SID number and receive any investigational product will be followed according to the protocol unless consent for follow-up is withdrawn. The investigator must notify the sponsor or designee of deviations from protocol visits or evaluations and these evaluations, if applicable, must be rescheduled or performed at the nearest possible time to the original schedule. Protocol deviations will be recorded on the

source document with an explanation for the deviation. The investigator must comply with the applicable requirements related to the reporting of protocol deviations to the IRB/IEC.

Subjects/legal representatives will be instructed to call study personnel to report any abnormalities during the intervals between study visits and to come to the study site if medical evaluation is needed and the urgency of the situation permits. For those subjects who experience a significant febrile illness within 7 days after receipt of the first dose of investigational product, a medical evaluation (to determine the probable etiology of the fever) is to be performed within 24 hours after the onset of fever, or as soon as possible thereafter. For emergency and other unscheduled visits to a medical facility other than the study site, medical records will be obtained by the investigator and made available to the sponsor or designee during monitoring visits.

A schedule of study procedures is presented in Table 5.2-1, followed by a description of each visit. A description of the study procedures is included in Section 5.3.

Table 5.2-1 Schedule of Evaluations

Study Period	Screen	Dose 1	Dose 1 Follow-up			Dose 2	Dose 2 Follow-up			End of Study
**Visit or Telephone Contact (TC)**	**Visit**	**Visit**	**Unscheduled Visit**	**TC**	**Visit**	**Visit**	**TC**	**Visit**	**Monthly TC**	**Final TC**
**Day**	**-14 to 1**	**1**	**2-8**	**2-8, 9, 10-16 [a]**	**15[b]**	**29 [c]**	**30-36, 37, 38-44 [a]**	**57 [c]**	**61, 91, 121, 151, 181[d]**	**209[e]**
**Interval (Days or Months Post Dose)**	**NA**	**NA**	**14 days**			**NA**	**14 days**	**NA**	**1, 2, 3, 4, 5 months**	**6 months post Dose 2**
**Procedure**										
Written informed consent	X									
Medical history, physical examination	X									
Update health status		X				X				
Record concomitant medications	X	X	X	X	X	X	X			
Pregnancy test, if needed	X	X				X				
Assess predose AEs, SAEs	X	X				X				

Table 5.2-1 Schedule of Evaluations

Study Period	Screen	Dose 1	Dose 1 Follow-up			Dose 2	Dose 2 Follow-up			End of Study
Visit or Telephone Contact (TC)	Visit	Visit	Unscheduled Visit	TC	Visit	Visit	TC	Visit	Monthly TC	Final TC
Day	-14 to 1	1	2-8	2-8, 9, 10-16 [a]	15[b]	29 [c]	30-36, 37, 38-44 [a]	57 [c]	61, 91, 121, 151, 181[d]	209[e]
Interval (Days or Months Post Dose)	NA	NA	14 days			NA	14 days	NA	1, 2, 3, 4, 5 months	6 months post Dose 2
**Procedure**										
Verify eligibility criteria	X	X				X				
Randomization using IVRS		X								
Collect blood for HAI testing		X			X	X		X		
Provide thermometers, memory aids		X				X				
**Administer investigational product**		**X**				**X**				
Collect post dose solicited		X	X	X	X	X	X			

Table 5.2-1 Schedule of Evaluations

Study Period	Screen	Dose 1	Dose 1 Follow-up			Dose 2	Dose 2 Follow-up			End of Study
Visit or Telephone Contact (TC)	Visit	Visit	Unscheduled Visit	TC	Visit	Visit	TC	Visit	Monthly TC	Final TC
Day	-14 to 1	1	2-8	2-8, 9, 10-16 [a]	15[b]	29 [c]	30-36, 37, 38-44 [a]	57 [c]	61, 91, 121, 151, 181[d]	209[e]
Interval (Days or Months Post Dose)	NA	NA	14 days			NA	14 days	NA	1, 2, 3, 4, 5 months	6 months post Dose 2
Procedure										
symptoms and AEs (through 14 days post dose)										
Collect post dose SAEs and NOCDs		X	X	X	X	X	X	X	X	X

[a] Telephone contacts every other day during the 7-day period post each dose (Days 2-8, Days 30-36), on the eighth day post each dose (Day 9, Day 37), and every other day for 9 to 14 days post each dose (Days 10-16, Days 38-44).

b The Day 15 visit (for subjects randomized to this visit) should occur 14 to 16 days post Dose 1 (Day 15-17, ie, Day 15 + 2).

[c] The Day 29 visit should occur 28 to 30 days post Dose 1(Day 29-31, ie, Day 29 + 2). The Day 57 visit should occur 28 to 30 days post Dose 2 (ie, from Days 57 to 61, depending on the timing of Dose 2).

[d] Monthly telephone contacts will be performed 1, 2, 3, 4, and 5 months post Dose 2, ie, on Days 61-68, 91-98, 121-128, 151-158, and 181-188.

e Final telephone contact will be performed on Day 209-219.

Note: Subjects randomized to placebo on Day 1 will be given the option of receiving 2 doses of approved product 28 days apart. The first optional dose of approved vaccine will be administered after the Day 57 immunogenicity blood samples are collected, and after FDA approves strain change (see Section 5.2.5.3).

5.2.1 Screening Visit (Day -14 to 1)

All screening procedures must be performed within 14 days prior to or on the same day as investigational product administration (Day -14 to Day 1). The screening evaluations may be carried out over more than one visit. Written informed consent and any locally required authorization (eg, HIPAA in the USA, EU Data Privacy Directive authorization in the EU) must be obtained prior to performing any study-related procedure, including screening evaluations.

1) Obtain written informed consent and appropriate privacy act document authorization (eg, HIPAA)
2) Assign an SID number using the IVRS
3) Obtain screening medical history
4) Perform screening physical exam, including oral temperature
5) Record concomitant medication use
6) Collect blood or urine sample for pregnancy test for females of childbearing potential (if screening and dosing occur at the same visit, only one test is required). A negative pregnancy test result must be confirmed before randomizing the subject.
7) Verify eligibility criteria

5.2.2 Dose 1 of Investigational Product (Day 1)

If screening and Day 1 procedures are performed on the same day, procedures completed for screening (eg, pregnancy testing) do not have to be repeated for Day 1. The following procedures will be done:

1) Record concomitant medications
2) Collect blood or urine sample for pregnancy test (for women of childbearing potential); result must be negative for randomization and dosing to occur
3) Assess for predose AEs (Section 6.1.1) and SAEs (Section 6.1.2) that may have occurred after written informed consent was obtained
4) Verify eligibility criteria
5) Contact IVRS to randomize and to assign investigational product kit number for subject
6) Collect blood sample for baseline HAI testing (prior to dosing)

7) Provide standardized thermometer, memory aid worksheets, and instructions for completing the worksheets to subject. Ensure that the subject understands how to take his/her oral temperature. The memory aid worksheets for collection of solicited symptoms, AEs, and concomitant medication use will be distributed with instructions; these worksheets will be completed by the subject to serve as a memory aid for future data collection by site personnel during telephone contacts. The worksheets will not be collected by site personnel.
8) Administer investigational product
9) Observe subject for a minimum of 15 minutes.
10) Record solicited symptoms, AEs, and concomitant medication use
11) Record any SAEs and NOCDs

5.2.3 Follow-up Period Post Dose 1 of Investigational Product

5.2.3.1 Unscheduled Visit for Significant Febrile Illness Through Day 8 (Days 2-8)

If a significant febrile illness occurs from Days 2 to 8 post Dose 1, a visit to perform a medical evaluation should occur:

1) Record solicited symptoms, AEs, and concomitant medication use
2) Record any SAEs and NOCDs
3) Perform a targeted medical history and physical examination.

5.2.3.2 Telephone Contacts During Days 2 to 16

Telephone contacts with the subject should be performed every other day during Days 2-8, on Day 9, and every other day during Days 10-16. At each phone contact the following will be done:

1) Record solicited symptoms, AEs, and concomitant medication use
2) Record any SAEs and NOCDs

5.2.3.3 Post Dose 1 Immunigenicity Blood Draw Visit (Day 15 + 2)

Based on randomization, selected subjects will return to the site for an immunogenicity blood draw 14-16 days post Dose 1 (ie, Day 15-17). The following will be performed at the Day 15 visit:

1) Record concomitant medications

2) Assess and record any AEs that have occurred since the last study contact
3) Assess and record and SAEs or NOCDs that may have occurred since the last study contact
4) Collect blood sample for HAI testing
5) Record solicited symptoms, AEs, and concomitant medication use

5.2.4 Dose 2 of investigational Product (Day 29 + 2)

All subjects will return to the site on Day 29. Subjects that were randomized to the post Dose 1 immunogenicity blood draw on Day 29 will have a predose blood draw to assess immune response. All subjects will receive the second dose of investigational product. The Day 29 visit should be performed 28-30 days post Dose 1, ie, from Days 29 to 31. The following will be done at the Day 29 visit:

1) Update health status
2) Record concomitant medications
3) Assess and record any AEs that have occurred since the last study contact with onset during the defined AE reporting period
4) Assess and record and SAEs or NOCDs that may have occurred since the last study contact
5) Collect blood sample for HAI testing
6) Collect blood or urine sample for pregnancy test. A negative pregnancy test result must be confirmed before administration of the second dose of investigational product is administered
7) Verify eligibility criteria
8) Contact IVRS to assign investigational product kit number for subject
9) Administer second dose of investigational product (according to the subject's original treatment assignment)
10) Observe subject for a minimum of 15 minutes.
11) Confirm that subject still has standardized thermometer provided on Day 1. Provide memory aid worksheets and instructions for completing the worksheets to the subject. Ensure that the subject understands how to take his/her oral temperature. The memory aid worksheets for collection of solicited symptoms, AEs, and concomitant medication use will be distributed with instructions; these worksheets will be completed by the subject to serve as a memory aid for future data collection by site personnel during telephone contacts. The worksheets will not be collected by site personnel.

12) Record solicited symptoms, AEs, and concomitant medication use.

5.2.5 Follow-up Period Post Dose 2 of Investigational Product

5.2.5.1 Telephone Contacts During 14 Days Post Dose 2

Telephone contacts with the subject should be performed every other day for safety assessments during the first 2 weeks after the second dose of investigational product. It should be noted that this 14-day interval will be in relation to the date on which the second dose occurs within the Day 29-31 dosing interval. At each phone contact the following will be done:

1) Record solicited symptoms, AEs, and concomitant medication use
2) Record any SAEs and NOCDs

5.2.5.2 Post Dose 2 Immunogenicity Blood Draw Visit (Day 57 + 4)

All subjects will return to the site for a final blood draw to assess immune response. This visit should be performed 28 to 30 days after receiving the second dose of investigational product (ie, from Days 57 to 61, depending on when Dose 2 was administered). The following will be performed:

1) Collect blood sample for HAI testing
2) Record any SAEs and NOCDs

5.2.5.3 Optional Vaccinations for Placebo Recipients to Receive Doses of Approved Product

After the strain-change supplement has been approved by FDA, subjects who were randomized to placebo will be offered the option of receiving dosing with approved product. For subjects who agree, two doses of the approved product will be administered by site personnel approximately 28 days apart. The date/time of dosing should be recorded in the subject's study records. It should be noted that the product will be approved, however the formulation provided will not be the commercial formulation; it will be the active monovalent vaccine described in Section 4.5.1.

The exact timing of this will depend on approval by the FDA. The first optional dose of approved product may not be administered until after the Day 57 blood sample for HAI testing has been collected (see Section 5.2.5.2). The following will be done:

1) Offer optional doses of approved product to subjects who were randomized to and received placebo on Day 1 and Day 29

2) If subject agrees, administer the first optional dose of unblinded active product. Observe subject for a minimum of 15 minutes post dose (see Section 6.4.3 regarding monitoring of dose administration)

3) Record any SAEs and NOCDs

4) Schedule the subject to return to the site in approximately 28 days to receive the second optional dose of unblinded active product. At that subsequent visit, observe the subject for a minimum of 15 minutes postdose and record any SAEs and NOCDs, as described above.

5.2.5.4 Monthly Telephone Contacts (Days 61, 91, 121, 151, and 181)

The telephone contacts at Days 61, 91, 121, 151, and 181 should be performed in relation to the Day 1 dose of investigational product. One telephone contact each will be performed from Days 61 to 68, Days 91 to 98, Days 121 to 128, Days 151 to 158, and Days 181 to 188. At each contact the following will be done:

- Monitor for SAEs and NOCDs

5.2.5.5 Final Study Telephone Contact (Days 209-219)

The final telephone contact should be performed at approximately 180 days following the second dose of investigational product. The following will be done:

- Monitor for SAEs and NOCDs through Day 209-211.

5.3 Description of Study Procedures

5.3.1 Medical History and Physical Examination

A medical history and physical examination including oral temperature will be performed at the screening visit.

5.3.2 Clinical Laboratory Tests

Serum or urine beta-human chorionic gonadotropin (ß-HCG) tests must be performed on any female subject of childbearing potential; a negative pregnancy test result must be confirmed before the subject is randomized and prior to receipt of Dose 2. Urine pregnancy tests may be performed at the site using a licensed test (dipstick).

5.3.3 Immunogenicity Evaluation and Methods

Individual subjects will have 3 blood samples collected for serum HAI testing: one on Day 1 after randomization but prior to administration of the first dose of investigational product, the second, based on randomization, on either the Day 15 or Day 29 visit (if Day 29, blood draw will occur prior to receiving the second dose of investigational product), and the third at the Day 57 visit, approximately 28 days after receipt of the second dose of investigational product. Serum will be separated and stored frozen at -20°C or below in a non-frost-free freezer until being batched and shipped to MedImmune (California) according to procedures specified in the Laboratory Manual.

Immune response evaluation will consist of serum HAI antibody titers to strains antigenically matched to the Influenza A (H1N1) 6:2 Virus Reassortant. Additional assays may be performed to investigate immune responses to influenza or other respiratory viruses. Samples may also be retained for potential future testing for immune response to influenza if the subject has given consent to potential future testing (no genetic testing will be performed). Immunogenicity assays will be conducted following standard assay procedures.

5.3.4 Estimate of Volume of Blood

A total maximum of approximately 20 to 25 mL of blood will be collected over the course of this study. Approximately 3 to 5 mL of blood will be collected for each of the 3 immunogenicity blood samples. Approximately 5 mL of blood will be collected for each pregnancy test if serum pregnancy testing is performed.

6 Assessment of Safety

6.1 Safety Parameters

6.1.1 Adverse Events

The ICH Guideline for Good Clinical Practice E6(R1) defines an adverse event (AE) as:

> Any untoward medical occurrence in a patient or clinical investigation subject administered a pharmaceutical product and which does not necessarily have a causal relationship with this treatment. An AE can therefore be any unfavorable and unintended sign (including an abnormal laboratory finding), symptom, or disease temporally associated with the use of a medicinal product, whether or not considered related to the medicinal product.

An AE includes but is not limited to any clinically significant worsening of a subject's pre-existing condition.

Adverse events may be treatment emergent (ie, occurring after initial receipt of investigational product) or nontreatment emergent. A nontreatment-emergent AE is any new sign or symptom, disease, or other untoward medical event that begins after the subject/legal representative signs the informed consent form but before the subject has received investigational product.

Elective treatment or surgery (that was scheduled prior to the subject being enrolled into the study) for a documented pre-existing condition that did not worsen from baseline is not considered an AE.

6.1.2 Serious Adverse Events

A serious adverse event (SAE) is any AE that:

- Results in death
- Is immediately life-threatening
- This term refers to an event in which the subject was at risk of death at the time of the event; it does not refer to an event that may have led to death.
- Requires inpatient hospitalization or prolongation of existing hospitalization

- In general, hospitalization signifies that the subject has been detained (usually involving at least an overnight stay) at the hospital or emergency ward for observation and/or treatment that would not have been appropriate in an outpatient setting.
- Results in persistent or significant disability/incapacity
- The term disability means a substantial disruption of a person’s ability to conduct normal life functions.
- Is a congenital anomaly/birth defect in offspring of the subject
- Is an important medical event that may jeopardize the subject or may require medical intervention to prevent one of the outcomes listed above. Medical or scientific judgment should be exercised in deciding whether expedited reporting is appropriate in this situation. Examples of medically important events are intensive treatment in an emergency room or at home for allergic bronchospasm, blood dyscrasias, or convulsions that do not result in hospitalizations; or development of drug dependency or drug abuse.

6.1.3 Solicited Symptoms

Solicited symptoms are events that are considered likely to occur post dosing. For this study, solicited symptoms include:

- Fever (> 100°F [37.8°C] oral)
- Runny nose
- Sore throat
- Cough
- Vomiting
- Muscle aches
- Chills
- Decreased activity (tiredness)
- Headache

6.1.4 New Onset Chronic Disease

An NOCD is a newly diagnosed medical condition that is of a chronic, ongoing nature and is assessed by the investigator as medically significant. Examples of NOCDs include but are not limited to diabetes, asthma, autoimmune disease (eg, lupus, rheumatoid arthritis), and neurological disease (eg, epilepsy, autism). Events that would not be considered NOCDs are mild eczema, diagnosis of a congenital anomaly present at study entry, or acute illness (eg,

otitis media, bronchitis). An NOCD should also be reported as an SAE if it meets the definition in Section 6.1.2. If an NOCD occurs within 14 days post dosing, the event should also be reported as an AE (Section 6.1.1).

6.2 Assessment and Recording of Safety Parameters

6.2.1 Assessment of Severity

Assessment of severity is one of the responsibilities of the investigator in the evaluation of AEs and SAEs. The determination of severity should be made by the investigator based upon medical judgment and the severity categories of Grade 1 to 5 as generally defined below.

Grade 1 An event that is usually transient and may require only minimal treatment or therapeutic intervention. The event does not generally interfere with usual activities of daily living.

Grade 2 An event that is usually alleviated with additional specific therapeutic intervention. The event interferes with usual activities of daily living, causing discomfort but poses no significant or permanent risk of harm to the subject.

Grade 3 An event that requires intensive therapeutic intervention. The event interrupts usual activities of daily living, or significantly affects the clinical status of the subject. The event poses a significant risk of harm to the subject, and hospitalization may be required.

Grade 4 An event, and/or its immediate sequelae, that is associated with an imminent risk of death or is with physical or mental disabilities that affect or limit the ability of the subject to perform activities of daily living (eating, ambulation, toileting, etc).

Grade 5 The termination of life as a result of an event.

It is important to distinguish between serious criteria and severity of an AE. Severity is a measure of intensity whereas seriousness is defined by the criteria in Section 6.1.2. A Grade 3 or Grade 4 AE need not necessarily be considered an SAE. For example, a Grade 3 headache that persists for several hours may not meet the regulatory definition of SAE criteria and would be considered a non-serious event, whereas a Grade 2 seizure resulting in a hospital admission would be considered an SAE.

6.2.2 Assessment of Relationship

An event is considered "product-related" for the purposes of regulatory reporting if the investigator, the MedImmune medical monitor, or the MedImmune Patient Safety Physician assesses the event as possibly, probably, or definitely related to the investigational product. This is not a conclusive determination of causal association between the product and the event.

Whenever the investigator's assessment is unknown or unclear, the event is treated as product-related for the purposes of reporting to regulatory authorities.

An event may be deemed to be not related to the product for purposes of regulatory reporting only if the investigator, MedImmune medical monitor, and MedImmune Patient Safety Physician, if applicable, agree that the event is not product-related.

The investigator is required to provide an assessment of relationship of AEs and SAEs to the investigational product. A number of factors should be considered in making this assessment including: 1) the temporal relationship of the event to the administration of investigational product; 2) whether an alternative etiology has been identified; and 3) biological plausibility. The following guidelines should be used by investigators to assess the relationship of an event to investigational product administration.

Relationship assessments that indicate an **"Unlikely Relationship"** to investigational product:

None: The event is related to an etiology other than the investigational product (the alternative etiology must be documented in the study subject's medical record).

Remote: The event is unlikely to be related to the investigational product and likely to be related to factors other than investigational product.

Relationship assessments that indicate a **"Likely Relationship"** to investigational product:

Possible: There is an association between the event and the administration of the investigational product, and there is a plausible mechanism for the event to be related to investigational product; but there may also be alternative etiology, such as characteristics of the subject's clinical status or underlying disease.

Probable: There is an association between the event and the administration of investigational product, a plausible mechanism for the event to be related to the investigational product, and the event could not be reasonably explained by known characteristics of the subject's clinical status or an alternative etiology is not apparent.

Definite: There is an association between the event and the administration of investigational product, a plausible mechanism for the event to be related to the investigational product, and causes other than the investigational product have been ruled out and/or the event re-appeared on re-exposure to the investigational product.

6.2.3 Recording of Adverse Events

Adverse events will be recorded on the CRF using a recognized medical term or diagnosis that accurately reflects the event. Adverse events will be assessed by the investigator for severity, relationship to the investigational product, possible etiologies, and whether the event meets criteria of an SAE and therefore requires immediate notification of the sponsor. See Section 6.1.2 for the definition of SAEs, and Section 6.2.1 and Section 6.2.2 for guidelines for assessment of severity and relationship, respectively. If an AE evolves into a condition that meets the regulatory definition of "serious," it will be reported on the SAE Report Form (Section 6.2.4).

6.2.4 Recording of Serious Adverse Events

Serious adverse events will be recorded on the SAE Report Form using a recognized medical term or diagnosis that accurately reflects the event. Serious adverse events will be assessed by the investigator for severity, relationship to the investigational product, and possible etiologies. See Section 6.1.2 for the definition of SAEs, and Section 6.2.1 and Section 6.2.2 regarding guidelines for assessment of severity and relationship, respectively.

For SAEs that occur prior to the administration of investigational product (nontreatment-emergent SAEs), an assessment of protocol relatedness must be made by the investigator. A protocol-related SAE may occur as a result of a procedure or intervention required during the screening process (eg, blood collection, washout of an existing medication) prior to the initial administration of investigational product. The following guidelines should be used by investigators to assess the relationship of nontreatment-emergent SAEs:

Protocol Related: The event occurred due to a procedure/intervention that was described in the protocol for which there is no alternative etiology present in the subject's medical record.

Not Protocol Related: The event is related to an etiology other than the procedure/ intervention that was described in the protocol (the alternative etiology must be documented in the study subject's medical record).

6.2.5 Recording of Solicited Symptoms

Solicited symptoms will be reported using the terms as defined in this protocol (see Section 6.1.3). Thermometers and safety assessment worksheets will be distributed to subjects on the day of investigational product administration. Beginning on the evening of the day of each investigational product administration, ie on Days 1 and 29, through 14 days after receipt of each dose of investigational product, the subject will record his/her oral temperature and the occurrence of solicited symptoms on the worksheets. Temperatures should be taken at approximately the same time each day (preferably in the evening); however, if multiple temperatures are taken during a given day, the highest temperature should be recorded on the worksheet regardless of the time of day taken.

6.2.6 Recording of New Onset Chronic Disease

NOCDs will be reported on the corresponding CRFs using a recognized medical term or diagnosis that accurately reflects the event.

6.3 Reporting Requirements for Safety Parameters

6.3.1 Study Reporting Period for Adverse Events

For subjects who are randomized and dosed in the study, the reporting period for AEs is the period immediately following the time that written informed consent is obtained through 14 days after each dose of investigational product (ie, Days 1-14 for Dose 1 and Days 29-43 for Dose 2). The subject will record any AEs from investigational product administration through 14 days after receipt of each dose of investigational product. Any AE that starts within the reporting period will be followed to resolution, even if the date extends beyond the

reporting period, up to the end of the clinical study. New onset (non-serious) AEs that present after the reporting period will not be collected.

6.3.2 Study Reporting Period for Serious Adverse Events

For subjects who are randomized and dosed in the study, the reporting period for SAEs is the period immediately following the time that written informed consent is obtained through 180 days after administration of the final dose of investigational product.

For subjects who are screen failures (ie, who are withdrawn from the study prior to randomization) or who are randomized but do not receive any dose of investigational product, the reporting period for SAEs is the period immediately following the time that written informed consent is obtained until the time that the subject is withdrawn from the study (ie, prior to randomization or Day 1 dose).

After submitting an initial SAE report for a subject (to MedImmune Patient Safety), the investigator is required to follow the subject proactively and provide further information on the subject's condition to MedImmune Patient Safety.

For all treatment-emergent SAEs, ie post dose SAEs occurring in subjects who received at least one dose of investigational product, the investigator is responsible for following all SAEs until resolution, even if this extends beyond the study reporting period of 180 days post final dose of investigational product, or until the subject returns to baseline status or the condition has stabilized with the expectation that it will remain chronic.

At any time after completion of the study, if an investigator or qualified designee becomes aware of an SAE that is suspected by the investigator or qualified designee to be related to investigational product, the event must be reported to MedImmune Patient Safety.

6.3.2.1 Notification of Sponsor of Serious Adverse Events

Within 24 hours of identifying an SAE, regardless of the presumed relationship to the investigational product, the investigator or qualified designee must complete the SAE Report Form and fax to MedImmune Patient Safety.

MedImmune Contact Information:

Patient Safety
MedImmune
One MedImmune Way
Gaithersburg, MD 20878
Fax: 1 301 398 4205

MedImmune, as sponsor of the study is responsible for reporting certain SAEs as expedited safety reports to applicable regulatory authorities, ethics committees, and participating investigators, in accordance with ICH Guidelines and/or local regulatory requirements. MedImmune may be required to report certain SAEs to regulatory authorities within 7 calendar days of being notified about the event; therefore, it is important that investigators submit additional information requested by MedImmune as soon as it becomes available.

Investigators should provide all available information at the time of SAE Report Form completion. Investigators should not wait to collect additional information to fully document the event before notifying MedImmune Patient Safety of an SAE. When additional information becomes available, submit a follow-up SAE Report Form (separate from the initial report form) with the new information. Any follow-up information to an SAE also needs to be provided to MedImmune Patient Safety within 24 hours of learning of the new information.

6.3.2.2 Notification of Institutional Review Board or Independent Ethics Committee of Serious Adverse Events

The investigator must comply with the applicable regulatory requirements related to the reporting of SAEs to the IRB/IEC. The IRB/IEC must be informed in a timely manner by the investigator of SAEs occurring at their site during the study. Investigators must also submit safety information provided by MedImmune to the IRB/IEC as detailed in Section 10.1 and Section 10.2.

6.3.3 Study Reporting Period for Solicited Symptoms

The reporting period for solicited symptoms for subjects is from the receipt of investigational product through 14 days after each of the two vaccinations (ie, Days 1-15 for Dose 1 and from Days 29-43 for Dose 2).

6.3.4 Study Reporting Period for New Onset of Chronic Disease

The reporting period for NOCDs is the period from the first dose of investigational product through 180 days after the second dose of investigational product.

6.3.5 Other Events Requiring Immediate Reporting

6.3.5.1 Pregnancy and Overdose

The following events are not necessarily considered to be AEs but are required to be reported ***within 24 hours of knowledge of the event*** to MedImmune Patient Safety using the Fax Notification Form (see contact information in Section 6.3.2.1):

1) Pregnancy (including the intention to become pregnant)
2) Investigational product overdose (whether or not the overdose is associated with an AE or SAE)

Subjects who are randomized, dosed, and then become pregnant during the study period must not receive additional doses of investigational product but will be followed for the duration of the study. A pregnancy should be followed for outcome and any premature terminations reported. In addition, the health status of the mother and child, including date of delivery, and the child's gender and weight should be reported to MedImmune Patient Safety after delivery.

6.3.5.2 Other Protocol-specific Events

The following events are also considered immediately reportable events and must be reported ***within 24 hours of knowledge of the event*** to MedImmune Patient Safety using the Fax Notification Form (see contact information in Section 6.3.2.1):

1) Any withdrawal of consent during the study
2) Any event resulting in discontinuation of investigational product (discontinuation of dosing is defined as lack of administration of the first dose to a subject who has been randomized or lack of administration of the second dose to a subject who received the first dose).

6.4 Safety Management During the Study

The MedImmune medical monitor has primary responsibility for the ongoing medical review of safety data throughout the study. This includes review of SAEs and timely review of AEs and "other events" reported during the study. MedImmune Patient Safety is responsible for the receipt, immediate review, investigation, and follow-up of SAEs reported from the clinical study sites.

The MedImmune Safety Monitoring Committee (SMC) will review safety data on a regular basis throughout the study and make recommendations regarding further conduct of the study. The MedImmune SMC may also review safety data at other time points in response to AEs assessed as medically relevant by the medical monitor.

6.4.1 Interruption or Permanent Discontinuation of Study Dosing in Individual Subjects

An individual subject will not receive any further investigational product if any of the following occur in the subject in question:

1. Withdrawal of consent
2. Event which, in the opinion of the investigator, contraindicates further dosing such as illnesses or complications
3. Fever ≥ 100.4°F (38.0°C) or evidence of significant active infection on the day of dosing
4. Anaphylactic reaction or other hypersensitivity reaction assessed as possibly, probably, or definitely related to investigational product by study investigator or medical monitor
5. Any known immunosuppressive condition or immune deficiency disease, including HIV infection, or ongoing immunosuppressive therapy
6. Receipt of any investigational drug therapy or expected receipt of investigational drug therapy within 30 days of Dose 2 (use of licensed agents for indications not listed in the package insert is permitted)
7. Use of antiviral therapy or antiviral agents with activity against influenza virus (including amantadine, rimantadine, oseltamivir, and zanamivir) within 48 hours prior to receipt of the second dose of investigational product or expected receipt of antiviral therapy or antiviral agents through 14 days after receipt second dose of investigational product
8. Receipt of any nonstudy vaccine within 30 days prior to Dose 2 or planned dosing with nonstudy vaccine through 30 days after Dose 2

9. A virologically confirmed case of influenza occurring prior to collection of the immunogenicity sample. Virologic confirmation is a positive result for influenza of any type in a laboratory assay of any type.

Note: A subject who initially is excluded from Dose 2 based on one or more of the above time-limited criteria (eg, fever) may be reconsidered for receipt of Dose 2 once the condition has resolved contingent that the subject does not meet any other criterion for temporary or permanent discontinuation of dosing and that dosing can occur within the required study window.

Subjects who are permanently discontinued from receiving investigational product will be followed for safety through the full study period (through 180 days post last dose), including the collection of any protocol-specified blood specimens, unless consent is withdrawn. Immunogenicity blood samples that are scheduled after a dose that was not received or after the occurrence of virologically confirmed influenza will not be obtained.

6.4.2 Study Stopping Criteria

If any of the following occur, administration of investigational product will be stopped and no additional subjects will be randomized into the study:

1) Death in any subject in which the cause of death is assessed as possibly, probably, or definitely related to investigational product
2) Anaphylactic reaction to investigational product in any subject
3) Immediately life-threatening reaction assessed as possibly, probably, or definitely related to investigational product

If any of the above-listed events occurs, a prompt cumulative review of safety data and the circumstances of the event in question will be conducted by the medical monitor, Patient Safety Physician, and the MedImmune SMC to determine whether dosing and study randomization should be resumed, whether the protocol will be modified, or whether the study will be discontinued permanently. Review and approval by the MedImmune SMC are required for resumption of the study in the event the study is interrupted because of one of the above-listed events. Where applicable, regulatory authorities and IRBs/IECs will be notified of any actions taken with the study.

Any subjects who have already received investigational product and are currently in the study at the time study stopping criteria are met will continue to be followed by the investigator for safety.

6.4.3 Monitoring of Dose Administration

After vaccination, all subjects will be observed for a minimum of 15 minutes by the study staff. Emergency management supplies (eg, AMBU bag, adrenaline [epinephrine], antihistamine) will remain available for initial treatment of an allergic reaction if needed. Local reactions or systemic events must be recorded on the CRF.

As with any vaccine, allergic reactions to dose administration are possible. Therefore, appropriate drugs and medical equipment to treat acute anaphylactic reactions must be immediately available, and study personnel must be trained to recognize and treat anaphylaxis.

7 Statistical Considerations

7.1 General Considerations

Data will be provided in data listings sorted by treatment group and subject number. Tabular summaries will be presented by treatment group. Categorical data will be summarized by the number and percentage of subjects in each category. Continuous variables will be summarized by descriptive statistics, including mean, standard deviation, median, minimum, and maximum. Confidence intervals (CIs) will be two-sided, unless otherwise stated.

7.2 Analysis Populations

The Intent-to-Treat (ITT) Population will include all randomized subjects.

The Safety Population includes all subjects who receive at least one dose of investigational product and experience any follow-up for safety. Treatment group will be assigned based on the actual study material received.

The Immunogenicity Population will be defined by dose number as specified below. In addition, protocol deviations will be reviewed by the study team. Subjects identified with deviations judged to potentially interfere with the generation or interpretation of an immune response will be excluded from the immunogenicity population. Treatment group will be assigned based on the actual study material received.

- Evaluable for post Dose 1 immunogenicity analysis: subjects who received Dose 1 of the study vaccine and have valid HAI measurements from blood samples obtained at baseline and post Dose 1
- Evaluable for post Dose 2 immunogenicity analysis: subjects who received 2 doses of the same study vaccine and have valid HAI measurements from blood samples obtained at baseline and post Dose 2

Missing data will be treated as missing; no data will be imputed.

7.3 Endpoints

7.3.1 Primary Endpoints

7.3.1.1 Primary Safety Endpoint

The primary safety endpoint of this study is fever post Dose 1 (Days 1-8) , defined as oral temperature ≥ 101°F (38.3°C). The rate of subjects with fever between the two treatment groups will be compared based on the upper limit of the two-sided 95% exact CIs for the rate difference (Vaccine minus Placebo). The upper limit of the two-sided 95% CI will be evaluated against the pre-specified equivalence criterion of 10% which corresponds to the following hypotheses:

H0 (null): Rate Difference ≥ 10%
HA (alternative): Rate Difference < 10%

A two-sided 95% CI will be constructed using the exact method based on the score statistic proposed by Chan and Zhang (Chan and Zhang, 1999). This method computes the lower and upper confidence limits by inverting two separate one-sided tests of half the nominal Type I error rate. Its test statistic is based on the score statistic that is computed as the observed difference of the two binomial event rates minus the hypothesized value of the rate difference divided by the standard error of this difference, with the standard error estimated by the constrained maximum likelihood method. This test assures that the Type I error does not exceed the prespecified level. Due to discreteness when the sample size is relatively small compared to the expected event rate, the associated Type I error rate could be quite conservative, that is, smaller than the pre-specified level.

7.3.1.2 Primary Immunogenicity Endpoint

The primary immunogenicity endpoint is the proportion of subjects who experience a post-dose seroresponse against the H1N1 strain in baseline seronegative subjects and in all subjects regardless of baseline serostatus, by dose number. Seroresponse is defined as a ≥ 4-fold rise in HAI titer from baseline.

For the immunogenicity analyses (including both primary and secondary endpoints), subjects with a strain-specific baseline HAI titer of ≤ 4 are considered to be seronegative to that strain; a value of 2 will be imputed for titers reported as < 4. All immunogenicity analyses will be based on the immunogenicity population.

7.3.2 Secondary Endpoints

7.3.2.1 Secondary Safety Endpoints

The secondary safety endpoints are:

- Other reported solicited symptoms, anti-pyretic and analgesic use, and AEs through 7 days after each vaccination with investigational product
- All solicited symptoms, anti-pyretic and analgesic use, and AEs through14 days after each vaccination with investigational product
- SAEs and NOCDs through 28 days after each vaccination with investigational product
- SAEs and NOCDs through 180 days after the final vaccination with investigational product

Fever will be summarized according to the following thresholds:

1) Oral > 100°F (37.8°C)
2) Oral ≥ 101°F (38.3°C)
3) Oral > 102°F (38.9°C)
4) Oral > 103°F (39.4°C)

Secondary analyses will be performed to compare rates between the two treatment groups of other solicited symptoms for the 7-day period following each dose. Exact two-sided 95% CIs (Chan and Zhang, 1999) on the rate difference (Vaccine minus Placebo) will be constructed. There are no pre-specified equivalence criteria for the secondary analyses. The number of days of solicited symptoms and the proportion of subjects experiencing each event by study day will be presented without formal statistical comparison. The distribution of the number of

days with each individual event will also be summarized without formal statistical comparison. The proportion of subjects using anti-pyretic and analgesic agents within 7 days after each vaccination and 14 days after each vaccination will be summarized by treatment group.

Adverse events, SAEs, and NOCDs will be summarized by system organ class and preferred term using the MedDRA dictionary.

7.3.2.2 Secondary Immunogenicity Endpoints

The secondary immunogenicity endpoints are:

- The proportion of subjects who achieve a post-dose HAI titer ≥ 32 against the H1N1 strain in baseline seronegative subjects and in all subjects regardless of baseline serostatus, by dose number.

- The serum HAI geometric mean titers (GMTs) in baseline seronegative subjects and in all subjects regardless of baseline serostatus, by dose number.

7.4 Planned Data Submissions

Five data submissions to the Center for Biologics Evaluation and Research (CBER) will be provided in the study. As described in Section 4.4, the study will be double-blind through Day 8 post Dose 1. An interim assessment of safety will be performed at Day 8 (7 days post Dose 1). To summarize the primary safety phase data (Days 1-8) and report the primary endpoint results to FDA, the study will be unblinded to MedImmune personnel at the Day 8 interim assessment. The Day 8 interim assessment will be conducted after the database is locked for Day 8 (post Dose 1) safety data and will include the primary safety endpoint of this study as well as analysis of other solicited symptoms, AEs, and antipyretic and analgesic use reported through Day 8.

Three subsequent planned interim submissions and a final report will be provided:

1) Dose 1 immunogenicity data through 14 days post Dose 1
2) Dose 1 safety and immunogenicity data through 28 days post Dose 1; this summary will also include preliminary Dose 2 safety data, ie, from the 7-day period post Dose 2
3) Dose 2 safety and immunogenicity data through 28 days post Dose 2

4) A final clinical study report of all safety data through 180 days post last dose will be provided after the study is complete and all data through the final visit are locked.

7.5 Sample Size and Power Calculations

The sample size and power calculations are based on the safety endpoints.

It is expected that almost all subjects will provide information on safety and tolerability. Table 7.5-1 summarizes the reactogenicity event rates following FluMist and placebo vaccination in healthy adults 18-64 years of age in Study AV009.

Table 7.5-1 Summary of Solicited Events (Days 0-7) in Study AV009

Number of Participants:	FluMist [a]	Placebo [a]
**Randomized**	**3041**	**1520**
**Who Returned Diary Cards**	**2985**	**1490**
**Who Experienced SEs**	**n (%)**	**n (%)**
Fever		
Oral Temperature > 100.0°F	40 (1.34)	20 (1.34)
Oral Temperature ≥ 101.0°F	17 (0.57)	9 (0.60)
Oral Temperature > 102.0°F	2 (0.07)	2 (0.13)
Oral Temperature > 103.0°F	0	0
Runny Nose	1323 (44.32)	397 (26.64)
Sore Throat	793 (26.57)	243 (16.31)
Cough	407 (13.64)	152 (10.20)
Headache	1172 (39.26)	555 (37.25)
Muscle Aches	481 (16.11)	216 (14.50)
Chills	247 (8.28)	91 (6.11)
Tired/Weak	733 (24.56)	306 (20.54)
Any Solicited Event	2117 (70.92)	919 (61.68)

SE = solicited event
[a] Participants counted if they experienced an event at least once within 7 days following vaccination.

With 300 evaluable subjects (240 vaccine and 60 placebo recipients), the power to rule out a fever rate difference of 10 percentage points between vaccine and placebo recipients for various true fever rates in placebo recipients (0.5% to 2.0%) is presented in Table 7.5-2. Accordingly, the currently proposed study will provide at least 99.9% power to rule out a rate

increase of 10 percentage points assuming the true difference between the treatment groups is zero and the true fever rate is ≤ 3%. Power is also high if the true difference is slightly greater than zero and the true fever rate is ≤ 3%. For example, if the true fever rates are 3% and 1% in vaccine and placebo recipients, respectively, the power will be 99.6%.

Table 7.5-2 Power to Rule Out a 10 Percentage Point Increase of Fever Rate in Vaccine Recipients

True Fever Rate		
**Vaccine (N=240)**	**Placebo (N=60)**	**Exact Power [a]**
0.5%	0.5%	>99.9%
1.0%	1.0%	>99.9%
1.5%	1.5%	>99.9%
2.0%	2.0%	>99.9%
2.0%	1.0%	>99.9%
3.0%	1.0%	99.6%

[a] Power was computed using the method proposed by Chan and Zhang (Chan and Zhang, 1999) and Proc-StatXact (Version 6.2) software.

Power to show similar event rates for other solicited symptoms with the currently proposed study design for rates ranging from 6% to 37% in placebo recipients is presented in Table 7.5-3. Accordingly, the currently proposed study provides at least 87% power to rule out a 20 percentage point increase, assuming the true difference between the two treatment groups is zero. Power is lower if the true difference is greater than zero.

Table 7.5-3 Power for Secondary Endpoints

Solicited Event	True Event Rate		PPT Increase to be Ruled Out	Power [a]
	Vaccine (N=240)	Placebo (N=60)		
Runny nose	27%	27%	20 PPT	94%
	44%	27%	20 PPT	8%
	44%	27%	36 PPT	90%
Sore throat	16%	16%	15 PPT	92%
	27%	16%	15 PPT	13%
	27%	16%	27 PPT	91%
Cough	10%	10%	15 PPT	99%
	14%	10%	15 PPT	82%
	14%	10%	17 PPT	93%
Headache	37%	37%	20 PPT	87%
Muscle aches	15%	15%	15 PPT	93%
Chills	6%	6%	10 PPT	95%
Tired/Weak	21%	21%	20 PPT	98%
	25%	21%	20 PPT	86%
	25%	21%	21 PPT	90%

PPT=percentage points
[a] Power was computed using the method proposed by Chan and Zhang (Chan and Zhang, 1999) and Proc-StatXact (Version 6.2) software.

7.6 Multiplicity Considerations

This study has a single hypothesis testing for the primary safety endpoint of fever (Days 1-8) after Dose 1, where a single confidence interval with a one-sided 2.5% Type I error rate will be constructed. Therefore, no multiplicity adjustment is necessary.

8 Direct Access to Source Data and Documents

The study will be monitored by MedImmune or its designee on a regular basis throughout the study period. During monitoring visits, the investigator will provide direct access to all source documentation relevant to the subject’s participation in the study. Source documentation includes, but is not limited to, the subject’s clinic and/or office chart, hospital

chart, informed consent forms, treatment notes, laboratory reports, pharmacy records, radiographs, and any other records maintained to conduct and evaluate the clinical study. The investigator must also ensure that direct access to study documents be made available for study-related audits, IRB/IEC review, or regulatory inspection.

9 Quality Control and Quality Assurance

9.1 Data Collection

As part of the responsibilities assumed by participating in the study, the investigator agrees to maintain adequate and accurate case histories for the subjects treated under this protocol. Case histories include CRFs and supporting data including, but not limited to, signed and dated informed consent forms, progress notes, hospital charts, nurse's notes, diary cards, laboratory reports, electrocardiogram strips, etc.

9.2 Study Monitoring

The primary source document for this study will be the subject's medical record. If separate research records are maintained by the investigator(s), both the medical record and the research records will be monitored/audited for the purposes of the study.

The investigator and institutions involved in the study will permit study-related monitoring and provide direct access to all study records and facilities. Adequate time and space for monitoring visits should be made by the investigator or other investigator site staff.

The monitor will visit study facilities at periodic intervals, in addition to maintaining necessary contact through telephone, e-mail, and letter. The monitor will assess subject enrollment and informed consent procedures; investigational product storage, dispensing, administration and accountability; compliance with protocol procedures; completeness and accuracy of data entered onto validated data collection instruments (paper CRF or electronic data screen) against original source documents; and the occurrence of AEs/SAEs. All aspects of the study will be carefully monitored for compliance with the protocol, applicable government regulations, GCP, and the site's standard operating procedures.

The monitor will discuss the conduct and progress of the study with the investigator and other site staff. The investigator must cooperate with the monitor to ensure that any problems noted in the course of the monitoring are resolved.

9.3 Audit and Inspection of the Study

During the conduct of the study, the sponsor or its representative may conduct audits of any data and facility participating in the study. The investigator and institutions involved in the study will permit such study-related audits and provide direct access to all study records and facilities. The investigator must maintain a comprehensive and centralized filing system of all study-related documentation that is suitable for inspection by the sponsor or its designated monitors, Quality Assurance auditors, or regulatory agency representatives. The investigator agrees to participate in audits conducted at a convenient time in a reasonable manner.

Government regulatory authorities may also perform inspections either during or after the study. In the event of an inspection by any regulatory authority, the investigator should promptly notify the sponsor. The investigator agrees to cooperate fully with inspections conducted by regulatory authorities and to allow representatives of the regulatory authority access to all study records. The investigator will forward to the sponsor a copy of any inspection records received.

10 Ethics

10.1 Regulatory Considerations

The study will be conducted in accordance with the ICH guidelines on GCP, the GCPs applicable to any region where the study is conducted, and the ethical principles set forth in the Declaration of Helsinki. GCP is defined as a standard for the design, conduct, performance, monitoring, auditing, recording, analysis, and reporting of clinical studies in a way that provides assurance that the data and reported results are credible and accurate, and that the rights, safety, and well-being of study subjects are protected.

Per GCP, the protocol will be reviewed and approved by the IRB or IEC of each participating center prior to study initiation. Serious adverse events regardless of causality will be reported to the sponsor and to the IRB/IEC, and the investigator will keep the IRB/IEC informed as to the progress of the study.

The investigator will explain the nature of the study and will inform the subject/legal representative that participation is voluntary and that the subject can withdraw or be withdrawn from the study at any time. Written informed consent will be obtained from each subject/legal representative prior to the screening procedures to determine if study eligibility

criteria are met. A copy of the signed consent form will be given to every subject/legal representative, and the original will be maintained with the subject's records.

10.2 Institutional Review Board or Independent Ethics Committee

A list of IRB/IEC members or a Statement of GCP Compliance should be obtained by the investigator and provided to the sponsor.

Any documents that the IRB/IEC may need to fulfill its responsibilities, such as protocol amendments, and information concerning subject recruitment, payment, or compensation procedures, or information from the sponsor will be submitted to the IRB/IEC. The IRB/IEC's written unconditional approval of the study protocol, the informed consent form(s), and any other written materials to be provided to subjects will be in the possession of the investigator and the sponsor before the study is initiated. The IRB/IEC's unconditional approval statement will be transmitted by the investigator to the sponsor prior to shipment of investigational product supplies to the site. This approval must refer to the study by exact protocol title and number, and should identify the documents reviewed and the date of review.

Protocol modifications or changes may not be initiated without prior written IRB/IEC approval except when necessary to eliminate immediate hazards to the subjects or when the change(s) involves only logistical or administrative aspects of the study. Such modifications will be submitted to the IRB/IEC and written verification that the modification was submitted should be obtained.

The IRB/IEC must be informed by the investigator of informed consent form changes or revisions of other documents originally submitted for review; serious and/or unexpected adverse experiences occurring during the study; new information that may affect adversely the safety of the subjects or the conduct of the study; an annual update and/or request for re-approval; and when the study has been completed.

10.3 Informed Consent

Freely given informed consent will be obtained and documented for all subjects under this protocol (or a subject's legally authorized representative, if the subject is unable to provide informed consent) in accordance with the ICH guidelines on GCP, the GCPs applicable to

any region where the study is conducted, and the ethical principles set forth in the Declaration of Helsinki.

Information should be given in both oral and written form, and subjects or their legal representatives must be given ample opportunity to inquire about details of the study. Subjects or their legal representatives must be informed of the following:

- The study involves research.
- The aims, expected benefits, possible risks (including a statement that the particular treatment or procedure may involve risks to the subject or the fetus of the subject, if the subject should become pregnant) that are currently unforeseeable.
- The study procedures to be followed and alternative treatment available to them. Subjects or their legal representatives must receive an explanation as to whether any compensation and any medical treatments are available if injury occurs and, if so, what they consist of, or where further information may be obtained.
- Who to contact for answers to any questions relating to the research project.
- Participation is voluntary and that they are free to withdraw from the study for any reason at any time, without penalty or loss of benefits to which they are otherwise entitled.
- The extent of the confidentiality of subject records must be defined, and subjects or their legal representatives must be informed that applicable data protection legislation will be complied with.
- The monitor(s), auditor(s), IRB/IEC members, and the regulatory authorities will be granted direct access to the subject's original medical records for verification of clinical study procedures and/or data, without violating the confidentiality of the subject, to the extent permitted by the applicable laws and regulations and that, by signing a written informed consent form, the subject or the subject's legally acceptable representative is authorizing such access.

The consent form generated by the investigator must be approved by the IRB/IEC and be acceptable to MedImmune. Consent forms must be written so as to be understood by the prospective subject/legal representative. Informed consent will be documented by the use of a written consent form approved by the IRB/IEC and signed and dated by the subject or the subject's legally authorized representative, and by the person who conducted the informed consent discussion. The signature confirms the consent is based on information that has been understood. Each subject's signed informed consent form must be kept on file by the investigator for possible inspection by regulatory authorities and/or MedImmune professional and regulatory compliance persons. The subject or the subject's legally acceptable representative should receive a copy of the signed and dated written informed consent form

and any other written information provided to the subject, and should receive copies of any signed and dated consent form updates and any amendments to the written information provided to subjects.

11 Data Handling and Record Keeping

To maintain confidentiality, all laboratory specimens, evaluation forms, reports, and other records transmitted outside the clinical site will be identified by a subject's SID or coded number and date of birth. All study records, source medical records, and code sheets or logs linking a subject's name to an SID number will be kept in a secure location. Study records such as CRFs may be maintained electronically and require the same security and confidentiality as paper. Clinical information will not be released without written permission of the subject/legal representative, except as specified in the informed consent form (eg, necessary for monitoring by regulatory authorities or the sponsor of the clinical study). The investigator must also comply with all applicable privacy regulations (eg, HIPAA 1996, EU Data Protection Directive 95/46/EC).

Study documents (including subject records, copies of data submitted to the sponsor, study notebook, and pharmacy records) must be kept secured in accordance with MedImmune policies and applicable regulatory requirements for a period of 2 years following the last regulatory authority approval of a marketing application of FluMist and until there are no pending or contemplated marketing applications, or for 2 years after centers have been notified that clinical development of FluMist has been discontinued, or as otherwise required by local requirements, whichever is longer. There may be other circumstances for which MedImmune is required to maintain study records and, therefore, MedImmune should be contacted prior to removing study records for any reason.

12 Financing and Insurance

Financing and insurance are addressed in the individual site contracts.

13 Publication Policy

Publication by the site of any data from this study must be carried out in accordance with the clinical study agreement.

14 References

Chan IS, Zhang Z. Test-based exact confidence intervals for the difference of two binomial proportions. Biometrics 1999;55:1202-09.

Centers for Disease Control and Prevention. Interim guidance on case definitions to be used for investigations of swine influenza A (H1N1) cases. Available at http://www.cdc.gov/swineflu/casedef_swineflu.htm, accessed 18Jun2009.

Centers for Disease Control and Prevention. Interim guidelines on antiviral recommendations for patients with confirmed or suspected swine influenza A (H1N1) virus infection and close contacts. Available at http://www.cdc.gov/h1n1flu/recommendations.htm, accessed 18Jun2009.

Centers for Disease Control and Prevention. Novel H1N1 Flu Situation Update, updated 12Jun2009. Available at http://www.cdc.gov/h1n1flu/update.htm, accessed on 18Jun2009.

Hoffmann E, Mahmood K, Chen Z, Yang CF, Spaete J, Greenberg HB, et al. Multiple gene segments control the temperature sensitivity and attenuation phenotypes of ca B/Ann Arbor/1/66. J Virol. 2005 Sep;79(17):11014-21.

Jin H, Lu B, Zhou H, Ma C, Zhao J, Yang CF, et al. Multiple amino acid residues confer temperature sensitivity to human influenza virus vaccine strains (FluMist) derived from cold-adapted A/Ann Arbor/6/60. Virology. 2003;306:18-24.

Murphy BR, Coelingh K. Principles underlying the development and use of live attenuated cold-adapted influenza A and B virus vaccines. Viral Immunol. 2002;15(2):295-323.

World Health Organization. Influenza-like illness in the United States and Mexico *in* Programmes and projects, disease outbreak news, epidemic and pandemic alert and response (EPR). 24Apr2009. Available at http://www.who.int/csr/don/2009_04_24/en/index.html, accessed 18Jun2009.

World Health Organization. Influenza A (H1N1) - update 50 in Programmes and projects, epidemic and pandemic alert and response (EPR), disease outbreak news, 17Jun2009. Available at http://www.who.int/csr/don/2009_06_17/en/index.html accessed 18Jun2009.

15 Summary Protocol Amendments and Administrative Changes to the Protocol

The original protocol dated 14May2009 was submitted to the FDA for review but was not distributed to any sites or IRBs; no subjects were enrolled under the original protocol. The protocol was revised on 22Jun2009 in response to feedback from FDA. Due to the extent of the study design changes, a revised protocol (Amendment 1) has been issued.

Protocol Amendment 1, 22Jun2009

Major changes to the study design include:

- Two doses instead of 1 dose of investigational product will be given, on Day 1 and Day 29
- A predose pregnancy test will be performed on females of childbearing potential before receipt of Dose 2 of investigational product (Day 29)
- Three blood samples for immune response will be collected: on Day 1 predose, on Day 15 for selected subjects, on Day 29 predose for selected subjects, and on Day 57
- Safety evaluations including solicited symptoms and AEs will be collected for the 14-day period after each dose of investigational product, ie, during Days 1-15 and Days 29-43, instead of Days 1-15. Memory aid worksheets will be dispensed at both the Day 1 and Day 29 visits.
- Telephone contacts will also be made every other day during Days 29-44, in addition to those specified during Days 1-16.
- The final study contact will be approximately 180 days after the final dose of investigational product.
- SAEs and NOCDs will be collected through 180 days after the final dose of investigational product.
- Five instead of 3 data submissions will be performed: at Day 8, Day 15, Day 29, Day 57, and at the end of the study.
- Subjects who were randomized to receive placebo investigational product will be offered two optional doses instead of one dose of approved product once the strain-change supplement has been approved by FDA. The two doses will be administered approximately 28 days apart.
- The total duration of the study is now approximately 7 to 8 months.

Appendix 1 FluMist Full Prescribing Information

HIGHLIGHTS OF PRESCRIBING INFORMATION
These highlights do not include all the information needed to use FluMist safely and effectively. See full prescribing information for FluMist.

FluMist® Influenza Virus Vaccine Live, Intranasal
Intranasal Spray
2008-2009 Formula
Initial U.S. Approval: 2003

----------------------------RECENT MAJOR CHANGES--------------------------

Indications and Usage (1)	9/2007
Dosage and Administration, Dosing Information (2.1)	9/2007
Warnings and Precautions (5)	9/2007

----------------------------INDICATIONS AND USAGE---------------------------

FluMist is a vaccine indicated for the active immunization of individuals 2-49 years of age against influenza disease caused by influenza virus subtypes A and type B contained in the vaccine. (1)

----------------------DOSAGE AND ADMINISTRATION-----------------------

For intranasal administration by a health care provider.

Age Group	Vaccination Status	Dosage Schedule
Children (2-8 years)	Not previously vaccinated with influenza vaccine	2 doses (0.2 mL* each, at least 1 month apart) (2.1)
Children (2-8 years)	Previously vaccinated with influenza vaccine	1 dose (0.2 mL*) (2.1)
Children, adolescents and adults (9-49 years)	Not applicable	1 dose (0.2 mL*) (2.1)

* Administer as 0.1 mL per nostril.

---------------------DOSAGE FORMS AND STRENGTHS---------------------

0.2 mL pre-filled, single-use intranasal spray (3)
Each 0.2 mL dose contains $10^{6.5\text{-}7.5}$ FFU (fluorescent focus units) of live attenuated influenza virus reassortants of each of the three strains for the 2008-2009 season: A/South Dakota/6/2007 (H1N1) (A/Brisbane/59/2007-like), A/Uruguay/716/2007 (H3N2) (A/Brisbane/10/2007-like), and B/Florida/4/2006. (3)

-------------------------------CONTRAINDICATIONS------------------------------

- Hypersensitivity to eggs, egg proteins, gentamicin, gelatin or arginine or life threatening reactions to previous influenza vaccination. (4.1)
- Concomitant aspirin therapy in children and adolescents. (4.2)

-----------------------WARNINGS AND PRECAUTIONS------------------------

- Do not administer FluMist to children <24 months of age because of increased risk of hospitalization and wheezing observed in clinical trials. (5.1)
- FluMist should not be administered to any individuals with asthma or children < 5 years of age with recurrent wheezing because of the potential for increased risk of wheezing post vaccination. (5.2)
- If Guillain-Barré syndrome has occurred with any prior influenza vaccination, the decision to give FluMist should be based on careful consideration of the potential benefits and risks. (5.3)
- Administration of FluMist, a live virus vaccine, to immunocompromised persons should be based on careful consideration of potential benefits and risks. (5.4)
- Safety has not been established in individuals with underlying medical conditions predisposing them to wild-type influenza infection complications. (5.5)

------------------------------ADVERSE REACTIONS-------------------------------

Most common adverse reactions (≥ 10% in FluMist and at least 5% greater than in control) are runny nose or nasal congestion in all ages, fever >100°F in children 2-6 years of age, and sore throat in adults. (6.1)

To report SUSPECTED ADVERSE REACTIONS, contact MedImmune at 1-877-633-4411 or VAERS at 1-800-822-7967 or *http://vaers.hhs.gov*.

------------------------------DRUG INTERACTIONS-------------------------------

- Antiviral agents active against influenza A and/or B: Do not administer FluMist until 48 hours after antiviral cessation. Antiviral agents should not be administered until 2 weeks after FluMist administration unless medically necessary. (7.2)

-----------------------USE IN SPECIFIC POPULATIONS------------------------

- Safety and effectiveness of FluMist have not been studied in pregnant women or nursing mothers. (8.1, 8.3)

See 17 for PATIENT COUNSELING INFORMATION.
Revised: 06/2008

FULL PRESCRIBING INFORMATION: CONTENTS*

1 INDICATIONS AND USAGE
2 DOSAGE AND ADMINISTRATION
2.1 Dosing Information
2.2 Administration Instructions
3 DOSAGE FORMS AND STRENGTHS
4 CONTRAINDICATIONS
4.1 Hypersensitivity
4.2 Concomitant Pediatric and Adolescent Aspirin Therapy and Reye's Syndrome
5 WARNINGS AND PRECAUTIONS
5.1 Risks in Children <24 Months of Age
5.2 Asthma/Recurrent Wheezing
5.3 Guillain-Barré Syndrome
5.4 Altered Immunocompetence
5.5 Medical Conditions Predisposing to Influenza Complications
5.6 Preventing and Managing Allergic Vaccine Reactions
5.7 Limitations of Vaccine Effectiveness
6 ADVERSE REACTIONS
6.1 Adverse Reactions in Clinical Trials
6.2 Postmarketing Experience
7 DRUG INTERACTIONS
7.1 Aspirin Therapy
7.2 Antiviral Agents Against Influenza A and/or B
7.3 Concomitant Inactivated Vaccines
7.4 Concomitant Live Vaccines
7.5 Intranasal Products
8 USE IN SPECIFIC POPULATIONS
8.1 Pregnancy
8.3 Nursing Mothers
8.4 Pediatric Use
8.5 Geriatric Use
8.6 Use in Individuals 50-64 Years of Age
11 DESCRIPTION
12 CLINICAL PHARMACOLOGY
12.1 Mechanism of Action
12.2 Biodistribution
13 NONCLINICAL TOXICOLOGY
13.1 Carcinogenesis, Mutagenesis, Impairment of Fertility
14 CLINICAL STUDIES
14.1 Studies in Children and Adolescents
14.2 Study in Adults
14.3 Study in Adults with HIV Infection
14.4 Refrigerated Formulation Study
14.5 Transmission Study
15 REFERENCES
16 HOW SUPPLIED/STORAGE AND HANDLING
17 PATIENT COUNSELING INFORMATION
17.1 Asthma and Recurrent Wheezing
17.2 Vaccination with Live Viral Vaccines
17.3 Adverse Event Reporting

*Sections or subsections omitted from the full prescribing information are not listed.

FULL PRESCRIBING INFORMATION

1 INDICATIONS AND USAGE

FluMist is a vaccine indicated for the active immunization of individuals 2-49 years of age against influenza disease caused by influenza virus subtypes A and type B contained in the vaccine.

2 DOSAGE AND ADMINISTRATION

FOR INTRANASAL ADMINISTRATION BY A HEALTH CARE PROVIDER.

2.1 Dosing Information

FluMist should be administered according to the following schedule:

Age Group	Vaccination Status	Dosage Schedule
Children age 2 years through 8 years	Not previously vaccinated with influenza vaccine	2 doses (0.2 mL* each, at least 1 month apart)
Children age 2 years through 8 years	Previously vaccinated with influenza vaccine	1 dose (0.2 mL*)
Children, adolescents and adults age 9 through 49 years	Not applicable	1 dose (0.2 mL*)

* Administer as 0.1 mL per nostril.

For children age 2 years through 8 years who have not previously received influenza vaccine, the recommended dosage schedule for nasal administration is one 0.2 mL dose (0.1 mL per nostril) followed by a second 0.2 mL dose (0.1 mL per nostril) given at least 1 month later.

For all other individuals, including children age 2-8 years who have previously received influenza vaccine, the recommended schedule is one 0.2 mL dose (0.1 mL per nostril).

FluMist should be administered prior to exposure to influenza. Annual revaccination with influenza vaccine is recommended.

2.2 Administration Instructions

Each sprayer contains a single dose of FluMist; approximately one-half of the contents should be administered into each nostril. 0.1 mL (i.e., half of the dose from a single FluMist sprayer) is administered into each nostril while the recipient is in an upright position. Insert the tip of the sprayer just inside the nose and rapidly depress the plunger until the dose-divider clip stops the plunger. The dose-divider clip is removed from the sprayer to administer the second half of the dose (0.1 mL) into the other nostril. Once FluMist has been administered, the sprayer should be disposed of according to the standard procedures for medical waste (e.g., sharps container or biohazard container).

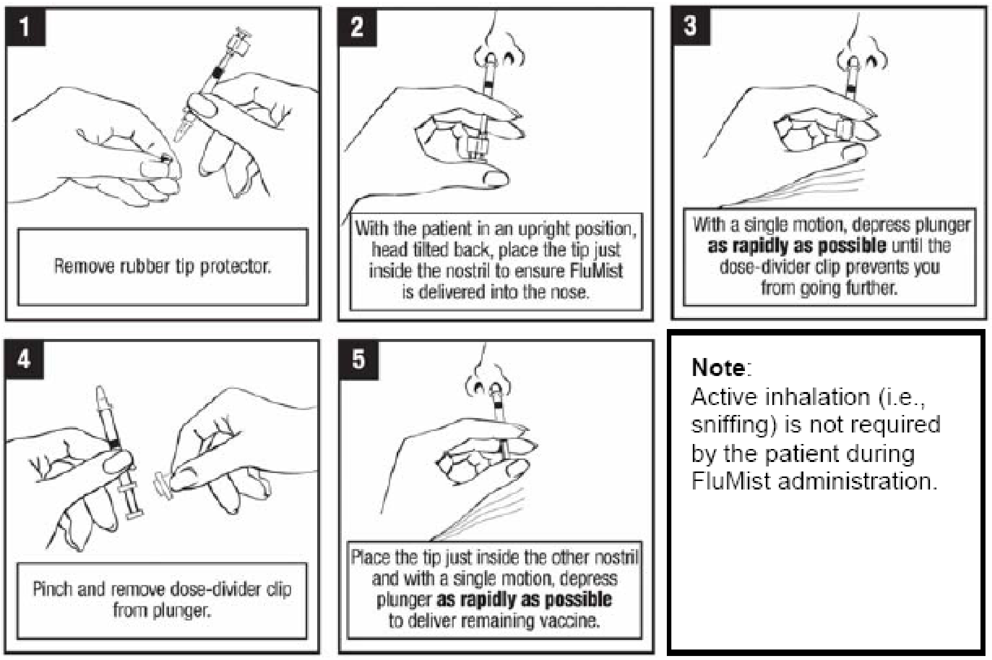

3 DOSAGE FORMS AND STRENGTHS

0.2 mL pre-filled, single-use intranasal spray.

Each 0.2 mL dose of FluMist is formulated to contain $10^{6.5\text{-}7.5}$ FFU (fluorescent focus units) of each of three live attenuated influenza virus reassortants: A/South Dakota/6/2007 (H1N1) (A/Brisbane/59/2007-like), A/Uruguay/716/2007 (H3N2) (A/Brisbane/10/2007-like), and B/Florida/4/2006 [1].

4 CONTRAINDICATIONS

4.1 Hypersensitivity

FluMist is contraindicated in individuals with a history of hypersensitivity, especially anaphylactic reactions, to eggs, egg proteins, gentamicin, gelatin, or arginine or with life-threatening reactions to previous influenza vaccinations.

4.2 Concomitant Pediatric and Adolescent Aspirin Therapy and Reye's Syndrome

FluMist is contraindicated in children and adolescents (2-17 years of age) receiving aspirin therapy or aspirin-containing therapy, because of the association of Reye's syndrome with aspirin and wild-type influenza infection.

5 WARNINGS AND PRECAUTIONS

5.1 Risks in Children <24 Months of Age

Do not administer FluMist to children <24 months of age. In clinical trials, an increased risk of wheezing post-vaccination was observed in FluMist recipients <24 months of age. An increase in hospitalizations was observed in children <24 months of age after vaccination with FluMist. [*See Adverse Reactions (6.1).*]

5.2 Asthma/Recurrent Wheezing

FluMist should not be administered to any individuals with asthma or children < 5 years of age with recurrent wheezing because of the potential for increased risk of wheezing post vaccination unless the potential benefit outweighs the potential risk.

Do not administer FluMist to individuals with severe asthma or active wheezing because these individuals have not been studied in clinical trials.

5.3 Guillain-Barré Syndrome

If Guillain-Barré syndrome has occurred within 6 weeks of any prior influenza vaccination, the decision to give FluMist should be based on careful consideration of the potential benefits and potential risks [*see also Adverse Reactions (6.2)*].

5.4 Altered Immunocompetence

Administration of FluMist, a live virus vaccine, to immunocompromised persons should be based on careful consideration of potential benefits and risks. Although FluMist was studied in 57 asymptomatic or mildly symptomatic adults with HIV infection *[see Clinical Studies (14.3)]*, data supporting the safety and effectiveness of FluMist administration in immunocompromised individuals are limited.

5.5 Medical Conditions Predisposing to Influenza Complications

The safety of FluMist in individuals with underlying medical conditions that may predispose them to complications following wild-type influenza infection has not been established. FluMist should not be administered unless the potential benefit outweighs the potential risk.

5.6 Management of Acute Allergic Reactions

Appropriate medical treatment and supervision must be available to manage possible anaphylactic reactions following administration of the vaccine *[see Contraindications (4.1)]*.

5.7 Limitations of Vaccine Effectiveness

FluMist may not protect all individuals receiving the vaccine.

6 ADVERSE REACTIONS

FluMist is not indicated in children <24 months of age. In a clinical trial, among children 6-23 months of age, wheezing requiring bronchodilator therapy or with significant respiratory symptoms occurred in 5.9% of FluMist recipients compared to 3.8% of active control (injectable influenza vaccine made by Sanofi Pasteur Inc.) recipients (Relative Risk 1.5, 95% CI: 1.2, 2.1). Wheezing was not increased in children ≥24 months of age.

Hypersensitivity, including anaphylactic reaction, has been reported post-marketing.

[See *Warnings and Precautions (5.1)* and *Adverse Reactions (6.1, 6.2)*.]

6.1 Adverse Reactions in Clinical Trials

Because clinical trials are conducted under widely varying conditions, adverse reaction rates observed in the clinical trials of a drug cannot be directly compared to rates in the clinical trials of another drug and may not reflect the rates observed in practice.

A total of 9537 children and adolescents 1-17 years of age and 3041 adults 18-64 years of age received FluMist in randomized, placebo-controlled Studies D153-P501, AV006, D153-P526, AV019 and AV009 described below. In addition, 4179 children 6-59 months of age received FluMist in Study MI-CP111, a randomized, active-controlled trial. Among pediatric FluMist recipients 6 months-17 years of age, 50% were female; in the study of adults, 55% were female. In MI-CP111, AV006, D153-P526, AV019 and AV009, subjects were White (71%), Hispanic (11%), Asian (7%), Black (6%), and Other (5%), while in D153-P501, 99% of subjects were Asian.

<u>Adverse Reactions in Children and Adolescents</u>

In a placebo-controlled safety study (AV019) conducted in a large Health Maintenance Organization (HMO) in children 1-17 years of age (n = 9689), an increase in asthma events, captured by review of diagnostic codes, was observed in children <5 years of age (Relative Risk 3.53, 90% CI: 1.1, 15.7). This observation was prospectively evaluated in Study MI-CP111.

In MI-CP111, an active-controlled study, increases in wheezing and hospitalization (for any cause) were observed in children <24 months of age, as shown in Table 1.

Table 1

Percentages of Children with Hospitalizations and Wheezing from MI-CP111

Adverse Reaction	Age Group	FluMist	Active Control[a]
Hospitalizations[b]	6-23 months (n = 3967)	4.2 %	3.2 %
	24-59 months (n= 4385)	2.1 %	2.5 %
Wheezing[c]	6-23 months (n = 3967)	5.9 %	3.8 %
	24-59 months (n = 4385)	2.1 %	2.5 %

[a] Injectable influenza vaccine made by Sanofi Pasteur Inc.
[b] From randomization through 180 days post last vaccination.
[c] Wheezing requiring bronchodilator therapy or with significant respiratory symptoms evaluated from randomization through 42 days post last vaccination.

Most hospitalizations observed were gastrointestinal and respiratory tract infections and occurred more than 6 weeks post vaccination. In post hoc analysis, rates of hospitalization in children 6-11 months of age (n = 1376) were 6.1% in FluMist recipients and 2.6% in active control recipients.

Table 2 shows an analysis of pooled solicited events, occurring in at least 1% of FluMist recipients and at a higher rate compared to placebo, post Dose 1 for Study D153-P501 and AV006 and solicited events post Dose 1 for Study MI-CP111. Solicited events were those about which parents/guardians were specifically queried after vaccination with FluMist. In these studies, solicited events were documented for 10 days post vaccination. Solicited events post Dose 2 for FluMist were similar to those post Dose 1 and were generally observed at a lower frequency.

Table 2
Summary of Solicited Events Observed within 10 Days after Dose 1 for Vaccine[a] and either Placebo or Active Control Recipients; Children 2-6 Years of Age

	D153-P501 & AV006		MI-CP111	
	FluMist N=876-1759[c]	Placebo N=424-1034[c]	FluMist N=2170[c]	Active Control[b] N=2165[c]
Event	%	%	%	%
Runny Nose/ Nasal Congestion	58	50	51	42
Decreased Appetite	21	17	13	12
Irritability	21	19	12	11
Decreased Activity (Lethargy)	14	11	7	6
Sore Throat	11	9	5	6
Headache	9	7	3	3
Muscle Aches	6	3	2	2
Chills	4	3	2	2
Fever				
100-101°F Oral	9	6	6	4
101-102°F Oral	4	3	4	3

a Frozen formulation used in AV006; Refrigerated formulation used in D153-P501 and MI-CP111.
b Injectable influenza vaccine made by Sanofi Pasteur Inc.
c Number of evaluable subjects (those who returned diary cards) for each event. Range reflects differences in data collection between the 2 pooled studies.

In clinical studies D153-P501 and AV006, other adverse reactions in children occurring in at least 1% of FluMist recipients and at a higher rate compared to placebo were: abdominal pain (2% FluMist vs. 0% placebo) and otitis media (3% FluMist vs. 1% placebo).

An additional adverse reaction identified in the active-controlled trial, MI-CP111, occurring in at least 1% of FluMist recipients and at a higher rate compared to active control was sneezing (2% FluMist vs. 1% active control).

In a separate trial (MI-CP112) that compared the refrigerated and frozen formulations of FluMist in children and adults 5-49 years of age, the solicited events and other adverse events were consistent with observations from previous trials. Fever of >103°F was observed in 1 to 2% of children 5-8 years of age.

In a separate placebo-controlled trial (D153-P526) using the refrigerated formulation in a subset of older children and adolescents 9-17 years of age who received one dose of FluMist, the solicited events and other adverse events were generally consistent with observations from previous trials. Abdominal pain was reported in 12% of FluMist recipients compared to 4% of placebo recipients and decreased activity was reported in 6% of FluMist recipients compared to 0% of placebo recipients.

Adverse Reactions in Adults

In adults 18-49 years of age in Study AV009, summary of solicited adverse events occurring in at least 1% of FluMist recipients and at a higher rate compared to placebo include runny nose (44% FluMist vs. 27% placebo), headache (40% FluMist vs. 38% placebo), sore throat (28% FluMist vs. 17% placebo), tiredness/weakness (26% FluMist vs. 22% placebo), muscle aches (17% FluMist vs. 15% placebo), cough (14% FluMist vs. 11% placebo), and chills (9% FluMist vs. 6% placebo).

In addition to the solicited events, other adverse reactions from Study AV009 occurring in at least 1% of FluMist recipients and at a higher rate compared to placebo were: nasal congestion (9% FluMist vs. 2% placebo) and sinusitis (4% FluMist vs. 2% placebo).

6.2 Postmarketing Experience

The following adverse reactions have been identified during postapproval use of FluMist. Because these reactions are reported voluntarily from a population of uncertain size, it is not always possible to reliably estimate their frequency or establish a causal relationship to vaccine exposure.

Congenital, familial and genetic disorder: Exacerbation of symptoms of mitochondrial encephalomyopathy (Leigh syndrome).

Gastrointestinal disorders: Nausea, vomiting, diarrhea

Immune system disorders: Hypersensitivity reactions (including anaphylactic reaction, facial edema and urticaria)

Nervous system disorders: Guillain-Barré syndrome, Bell's Palsy

Respiratory, thoracic and mediastinal disorders: Epistaxis

Skin and subcutaneous tissue disorders: Rash

7 DRUG INTERACTIONS

7.1 Aspirin Therapy

Do not administer FluMist to children or adolescents who are receiving aspirin therapy or aspirin-containing therapy *[see Contraindications (4.2)]*.

7.2 Antiviral Agents Against Influenza A and/or B

The concurrent use of FluMist with antiviral agents that are active against influenza A and/or B viruses has not been evaluated. However, based upon the potential for antiviral agents to reduce the effectiveness of FluMist, do not administer FluMist until 48 hours after the cessation of antiviral therapy and antiviral agents should not be administered until two weeks after administration of FluMist unless medically indicated. If antiviral agents and FluMist are administered concomitantly, revaccination should be considered when appropriate.

7.3 Concomitant Inactivated Vaccines

The safety and immunogenicity of FluMist when administered concurrently with inactivated vaccines have not been determined. Studies of FluMist excluded subjects who received any inactivated or subunit vaccine within two weeks of enrollment. Therefore, healthcare providers should consider the risks and benefits of concurrent administration of FluMist with inactivated vaccines.

7.4 Concomitant Live Vaccines

Concurrent administration of FluMist with the measles, mumps and rubella vaccine and the varicella vaccine was studied in 1245 children 12-15 months of age. Adverse events were similar to those seen in other clinical trials with FluMist *[see Adverse Reactions (6.1)]*. No evidence of interference with immune responses to measles, mumps, rubella, varicella and FluMist vaccines was observed. The safety and immunogenicity in children >15 months of age have not been studied.

7.5 Intranasal Products

There are no data regarding co-administration of FluMist with other intranasal preparations.

8 USE IN SPECIFIC POPULATIONS

8.1 Pregnancy

Pregnancy Category C

Animal reproduction studies have not been conducted with FluMist. It is not known whether FluMist can cause fetal harm when administered to a pregnant woman or can affect reproduction capacity. FluMist should be given to a pregnant woman only if clearly needed.

The effect of the vaccine on embryo-fetal and pre-weaning development was evaluated in a developmental toxicity study using pregnant rats receiving the frozen formulation. Groups of animals were administered the vaccine either once (during the period of organogenesis on gestation day 6) or twice (prior to gestation and during the period of organogenesis on gestation

day 6), 250 microliter/rat/occasion (approximately 110-140 human dose equivalents), by intranasal instillation. No adverse effects on pregnancy, parturition, lactation, embryo-fetal or pre-weaning development were observed. There were no vaccine related fetal malformations or other evidence of teratogenesis noted in this study.

8.3 Nursing Mothers

It is not known whether FluMist is excreted in human milk. Therefore, as some viruses are excreted in human milk and additionally, because of the possibility of shedding of vaccine virus and the close proximity of a nursing infant and mother, caution should be exercised if FluMist is administered to nursing mothers.

8.4 Pediatric Use

Safety and effectiveness of the vaccine has been demonstrated for children 2 years of age and older with reduction in culture-confirmed influenza rates compared to active control (injectable influenza vaccine made by Sanofi Pasteur Inc.) and placebo *[see Clinical Studies (14.1)]*. FluMist is not indicated for use in children <24 months of age. FluMist use in children <24 months has been associated with increased risk of hospitalization and wheezing in clinical trials *[see Warnings and Precautions (5.1)* and *Adverse Reactions (6.1)]*.

8.5 Geriatric Use

FluMist is not indicated for use in individuals ≥65 years of age. Subjects with underlying high-risk medical conditions (n=200) were studied for safety. Compared to controls, FluMist recipients had a higher rate of sore throat.

8.6 Use in Individuals 50-64 Years of Age

FluMist is not indicated for use in individuals 50-64 years of age. In Study AV009, effectiveness was not demonstrated in individuals 50-64 years of age (n=641). Solicited adverse events were similar in type and frequency to those reported in younger adults.

11 DESCRIPTION

FluMist (Influenza Virus Vaccine Live, Intranasal) is a live trivalent vaccine for administration by intranasal spray. The influenza virus strains in FluMist are (a) *cold-adapted* (*ca*) (i.e., they replicate efficiently at 25°C, a temperature that is restrictive for replication of many wild-type influenza viruses); (b) *temperature-sensitive (ts)* (i.e., they are restricted in replication at 37°C (Type B strains) or 39°C (Type A strains), temperatures at which many wild-type influenza viruses grow efficiently); and (c) *attenuated (att)* (they do not produce classic influenza-like illness in the ferret model of human influenza infection). The cumulative effect of the antigenic properties and

the *ca*, *ts*, and *att* phenotypes is that the attenuated vaccine viruses replicate in the nasopharynx to induce protective immunity.

No evidence of reversion has been observed in the recovered vaccine strains that have been tested (135 of possible 250 recovered isolates) *[see Clinical Studies (14.5)]*. For each of the three reassortant strains in FluMist, the six internal gene segments responsible for *ca*, *ts*, and *att* phenotypes are derived from a master donor virus (MDV), and the two segments that encode the two surface glycoproteins, hemagglutinin (HA) and neuraminidase (NA), are derived from the corresponding antigenically relevant wild-type influenza viruses that have been recommended by the USPHS for inclusion in the annual vaccine formulation. Thus, the three viruses contained in FluMist maintain the replication characteristics and phenotypic properties of the MDV and express the HA and NA of wild-type viruses that are related to strains expected to circulate during the 2008-2009 influenza season. For the Type A MDV, at least five genetic loci in three different internal gene segments contribute to the *ts* and *att* phenotypes. For the Type B MDV, at least three genetic loci in two different internal gene segments contribute to both the *ts* and *att* properties; five genetic loci in three gene segments control the *ca* property.

Specific pathogen-free (SPF) eggs are inoculated with each of the reassortant strains and incubated to allow vaccine virus replication. The allantoic fluid of these eggs is harvested, pooled and then clarified by filtration. The virus is concentrated by ultracentrifugation and diluted with stabilizing buffer to obtain the final sucrose and potassium phosphate concentrations. Ethylene diamine tetracetic acid (EDTA) is added to the dilution buffer for H3N2 strains. The viral harvests are then sterile filtered to produce the monovalent bulks. Each lot is tested for *ca*, *ts*, and *att* phenotypes and is also tested extensively by *in vitro* and *in vivo* methods to detect adventitious agents. Monovalent bulks from the three strains are subsequently blended and diluted as required to attain the desired potency with stabilizing buffers to produce the trivalent bulk vaccine. The bulk vaccine is then filled directly into individual sprayers for nasal administration.

Each pre-filled refrigerated FluMist sprayer contains a single 0.2 mL dose. Each 0.2 mL dose contains $10^{6.5-7.5}$ FFU of live attenuated influenza virus reassortants of each of the three strains: A/South Dakota/6/2007 (H1N1) (A/Brisbane/59/2007-like), A/Uruguay/716/2007 (H3N2) (A/Brisbane/10/2007-like), and B/Florida/4/2006 [1]. Each 0.2 mL dose also contains 0.188 mg/dose monosodium glutamate, 2.00 mg/dose hydrolyzed porcine gelatin, 2.42 mg/dose arginine, 13.68 mg/dose sucrose, 2.26 mg/dose dibasic potassium phosphate, 0.96 mg/dose monosodium phosphate, and <0.015 mcg/mL gentamicin sulfate. FluMist contains no preservatives.

The tip attached to the sprayer is equipped with a nozzle that produces a fine mist that is primarily deposited in the nose and nasopharynx. FluMist is a colorless to pale yellow liquid and is clear to slightly cloudy.

12 CLINICAL PHARMACOLOGY

12.1 Mechanism of Action

Immune mechanisms conferring protection against influenza following receipt of FluMist vaccine are not fully understood. Likewise, naturally acquired immunity to wild-type influenza has not been completely elucidated. Serum antibodies, mucosal antibodies and influenza-specific T cells may play a role in prevention and recovery from infection.

Influenza illness and its complications follow infection with influenza viruses. Global surveillance of influenza identifies yearly antigenic variants. For example, since 1977, antigenic variants of influenza A (H1N1 and H3N2) viruses and influenza B viruses have been in global circulation. Antibody against one influenza virus type or subtype confers limited or no protection against another. Furthermore, antibody to one antigenic variant of influenza virus might not protect against a new antigenic variant of the same type or subtype. Frequent development of antigenic variants through antigenic drift is the virologic basis for seasonal epidemics and the reason for the usual change of one or more new strains in each year's influenza vaccine. Therefore, influenza vaccines are standardized to contain the strains (i.e., typically two type A and one type B), representing the influenza viruses likely to be circulating in the United States in the upcoming winter.

Annual revaccination with the current vaccine is recommended because immunity declines during the year after vaccination, and because circulating strains of influenza virus change from year to year.

12.2 Biodistribution

A biodistribution study of intranasally administered radiolabeled placebo was conducted in 7 healthy adult volunteers. The mean percentage of the delivered doses detected were as follows: nasal cavity 89.7%, stomach 2.6%, brain 2.4%, and lung 0.4%. The clinical significance of these findings is unknown.

13 NONCLINICAL TOXICOLOGY

13.1 Carcinogenesis, Mutagenesis, Impairment of Fertility

FluMist has not been evaluated for its carcinogenic or mutagenic potential or its potential to impair fertility.

14 CLINICAL STUDIES

FluMist, in refrigerated and frozen formulations, was administered to approximately 35,000 subjects in controlled clinical studies. FluMist has been studied in placebo-controlled trials over

multiple years, using different vaccine strains. Comparative efficacy has been studied where FluMist was compared to an inactivated influenza vaccine made by Sanofi Pasteur Inc.

14.1 Studies in Children and Adolescents

Study MI-CP111: Pediatric Comparative Study

A multinational, randomized, double-blind, active-controlled trial (MI-CP111) was performed to assess the efficacy and safety of FluMist compared to an injectable influenza vaccine made by Sanofi Pasteur Inc. (active control) in children <5 years of age, using the refrigerated formulation. During the 2004-2005 influenza season, a total number of 3916 children <5 years of age and without severe asthma, without use of bronchodilator or steroids and without wheezing within the prior 6 weeks were randomized to FluMist and 3936 were randomized to active control. Participants were then followed through the influenza season to identify illness caused by influenza virus. As the primary endpoint, culture-confirmed modified CDC-ILI (CDC-defined influenza-like illness) was defined as a positive culture for a wild-type influenza virus associated within ±7 days of modified CDC-ILI. Modified CDC-ILI was defined as fever (temperature ≥100°F oral or equivalent) plus cough, sore throat, or runny nose/nasal congestion on the same or consecutive days.

In the primary efficacy analysis, FluMist demonstrated a 44.5% (95% CI: 22.4, 60.6) reduction in influenza rate compared to active control as measured by culture-confirmed modified CDC-ILI caused by wild-type strains antigenically similar to those contained in the vaccine. See Table 3 for a description of the results by strain and antigenic similarity.

Table 3
Comparative Efficacy against Culture-Confirmed Modified CDC-ILI[a] Caused by Wild-Type Strains in Children <5 Years of Age

	FluMist			Active Control[b]			% Reduction in Rate for FluMist[c]	95% CI
	N	# of Cases	Rate (cases/N)	N	# of Cases	Rate (cases/N)		
Matched Strains								
All strains	3916	53	1.4%	3936	93	2.4%	44.5%	22.4, 60.6
A/H1N1	3916	3	0.1%	3936	27	0.7%	89.2%	67.7, 97.4
A/H3N2	3916	0	0.0%	3936	0	0.0%	--	--
B	3916	50	1.3%	3936	67	1.7%	27.3%	-4.8, 49.9
Mismatched Strains								
All strains	3916	102	2.6%	3936	245	6.2%	58.2%	47.4, 67.0
A/H1N1	3916	0	0.0%	3936	0	0.0%	--	--
A/H3N2	3916	37	0.9%	3936	178	4.5%	79.2%	70.6, 85.7
B	3916	66	1.7%	3936	71	1.8%	6.3%	-31.6, 33.3
Regardless of Match								
All strains	3916	153	3.9%	3936	338	8.6%	54.9%	45.4, 62.9
A/H1N1	3916	3	0.1%	3936	27	0.7%	89.2%	67.7, 97.4
A/H3N2	3916	37	0.9%	3936	178	4.5%	79.2%	70.6, 85.7
B	3916	115	2.9%	3936	136	3.5%	16.1%	-7.7, 34.7

ATP Population.

[a] Modified CDC-ILI was defined as fever (temperature ≥100°F oral or equivalent) plus cough, sore throat, or runny nose/nasal congestion on the same or consecutive days.
[b] Injectable influenza vaccine made by Sanofi Pasteur Inc.
[c] Reduction in rate was adjusted for country, age, prior influenza vaccination status, and wheezing history status.

Study D153-P501: Pediatric Study

A randomized, double-blind, placebo-controlled trial (D153-P501) was performed to evaluate the efficacy of FluMist in children 12 to 35 months of age without high-risk medical conditions against culture-confirmed influenza illness, using the refrigerated formulation. A total of 3174 children were randomized 3:2 (vaccine:placebo) to receive 2 doses of study vaccine or placebo at least 28 days apart in Year 1. See Table 4 for a description of the results.

Study AV006: Pediatric Study

AV006 was a multi-center, randomized, double-blind, placebo-controlled trial performed in U.S. children without high-risk medical conditions to evaluate the efficacy of FluMist against culture-confirmed influenza over two successive seasons using the frozen formulation. The primary endpoint of the trial was the prevention of culture-confirmed influenza illness due to antigenically matched wild-type influenza in children, who received two doses of vaccine in the first year and a single revaccination dose in the second year. During the first year of the study 1602 children 15-71 months of age were randomized 2:1 (vaccine:placebo). Approximately 85% of the participants in the first year returned for the second year of the study. In Year 2, children remained in the same treatment group as in year one and received a single dose of FluMist or placebo. See Table 4 for a description of the results.

Table 4
D153-P501 & AV006, Years 1[a]: Efficacy of FluMist vs. Placebo against Culture-Confirmed Influenza Illness due to Wild-Type Strains

	D153-P501			AV006		
	FluMist n[b] (%)	Placebo n[b] (%)	% Efficacy (95% CI)	FluMist n[b] (%)	Placebo n[b] (%)	% Efficacy (95% CI)
	N[c]=1653	N[c]=1111		N[c]=849	N[c]=410	
Any strain	56 (3.4%)	139 (12.5%)	72.9%[d] (62.8, 80.5)	10 (1%)	73 (18%)	93.4% (87.5, 96.5)
A/H1N1	23 (1.4%)	81 (7.3%)	80.9% (69.4, 88.5)[e]	0	0	--
A/H3N2	4 (0.2%)	27 (2.4%)	90.0% (71.4, 97.5)	4 (0.5%)	48 (12%)	96.0% (89.4, 98.5)
B	29 (1.8%)	35 (3.2%)	44.3% (6.2, 67.2)	6 (0.7%)	31 (7%)	90.5% (78.0, 95.9)

[a] D153-P501 and AV006 data are for subjects who received two doses of study vaccine.
[b] Number and percent of subjects in per-protocol efficacy analysis population with culture-confirmed influenza illness.
[c] Number of subjects in per-protocol efficacy analysis population of each treatment group of each study for the "any strain" analysis.
[d] For D153-P501, influenza circulated through 12 months following vaccination.
[e] Estimate includes A/H1N1 and A/H1N2 strains. Both were considered antigenically similar to the vaccine.

During the second year of Study AV006, the primary circulating strain was the A/Sydney/05/97 H3N2 strain, which was antigenically dissimilar from the H3N2 strain represented in the vaccine,

A/Wuhan/359/95; FluMist demonstrated 87.0% (95% CI: 77.0, 92.6) efficacy against culture-confirmed influenza illness.

14.2 Study in Adults

AV009 was a multi-center, randomized, double-blind, placebo-controlled trial to evaluate effectiveness in adults 18-64 years of age without high-risk medical conditions. Participants were randomized 2:1, vaccine:placebo. Cultures for influenza virus were not obtained from subjects in the trial, so that the efficacy against culture-confirmed influenza was not assessed. The A/Wuhan/359/95 (H3N2) strain, which was contained in FluMist, was antigenically distinct from the predominant circulating strain of influenza virus during the trial period, A/Sydney/05/97 (H3N2). Type A/Wuhan (H3N2) and Type B strains also circulated in the U.S. during the study period. The primary endpoint of the trial was the reduction in the proportion of participants with one or more episodes of any febrile illness and prospective secondary endpoints were severe febrile illness, and febrile upper respiratory illness. Effectiveness for any of the three endpoints was not demonstrated in a subgroup of adults 50-64 years of age. Primary and secondary effectiveness endpoints from the age group 18-49 years of age are presented in Table 5. Effectiveness was not demonstrated for the primary endpoint in adults 18-49 years of age.

Table 5
Effectiveness of FluMist[a] in Adults 18–49 Years of Age During the 7-week Site-Specific Outbreak Period

Endpoint	**FluMist** N=2411[b] n (%)	**Placebo** N=1226[b] n (%)	**Percent Reduction**	**(95% CI)**
**Participants with one or more events of:[c]**				
Primary Endpoint:				
Any febrile illness	331 (13.73)	189 (15.42)	**10.9**	(-5.1, 24.4)
Secondary Endpoints:				
Severe febrile illness	250 (10.37)	158 (12.89)	**19.5**	(3.0, 33.2)
Febrile upper respiratory illness	213 (8.83)	142 (11.58)	**23.7**	(6.7, 37.5)

[a] Frozen formulation used.
[b] Number of evaluable subjects (92.7% and 93.0% of FluMist and placebo recipients, respectively).
[c] The predominantly circulating virus during the trial period was A/Sydney/05/97 (H3N2), an antigenic variant not included in the vaccine.

Effectiveness was shown in a post-hoc analysis using CDC-ILI in the age group 18-49 years.

14.3 Study in Adults with Human Immunodeficiency Virus (HIV) Infection

Safety and shedding of vaccine virus following FluMist administration were evaluated in 57 HIV-infected [median CD4 cell count of 541 cells/mm^3] and 54 HIV-negative adults 18-58 years of age

in a randomized, double-blind, placebo controlled trial using the frozen formulation. No serious adverse events were reported during the one-month follow-up period. Vaccine strain (type B) virus was detected in 1 of 28 HIV-infected subjects on Day 5 only and none of the HIV-negative FluMist recipients. No adverse effects on HIV viral load or CD4 counts were identified following FluMist. The effectiveness of FluMist in preventing influenza illness in HIV-infected individuals has not been evaluated.

14.4 Refrigerated Formulation Study

A double-blind, randomized multi-center trial was conducted to evaluate the comparative immunogenicity and safety of refrigerated and frozen formulations of FluMist in individuals 5 to 49 years of age without high risk medical conditions. Nine hundred and eighty-one subjects were randomized at a 1:1 ratio to receive either vaccine formulation. Subjects 5-8 years of age received two doses of study vaccine 46-60 days apart; subjects 9-49 years of age received one dose of study vaccine. The study met its primary endpoint. The GMT ratios of refrigerated and frozen formulations (adjusted for baseline serostatus) for H1N1, H3N2 and B strains, respectively, were 1.24, 1.02 and 1.00 in the two dose group and 1.14, 1.12 and 0.96 in the one dose group.

14.5 Transmission Study

FluMist contains live attenuated influenza viruses that must infect and replicate in cells lining the nasopharynx of the recipient to induce immunity. Vaccine viruses capable of infection and replication can be cultured from nasal secretions obtained from vaccine recipients. The relationship of viral replication in a vaccine recipient and transmission of vaccine viruses to other individuals has not been established.

Using the frozen formulation, a prospective, randomized, double-blind, placebo-controlled trial was performed in a daycare setting in children <3 years of age to assess the transmission of vaccine viruses from a vaccinated individual to a non-vaccinated individual. A total of 197 children 8-36 months of age were randomized to receive one dose of FluMist (n=98) or placebo (n=99). Virus shedding was evaluated for 21 days by culture of nasal swab specimens. Wild-type A (H3N2) influenza virus was documented to have circulated in the community and in the study population during the trial, whereas Type A (H1N1) and Type B strains did not.

At least one vaccine strain was isolated from 80% of FluMist recipients; strains were recovered from 1-21 days post vaccination (mean duration of 7.6 days ± 3.4 days). The cold-adapted *(ca)* and temperature-sensitive (*ts)* phenotypes were preserved in 135 tested of 250 strains isolated at the local laboratory. Ten influenza isolates (9 influenza A, 1 influenza B) were cultured from a total of seven placebo subjects. One placebo subject had mild symptomatic Type B virus infection confirmed as a transmitted vaccine virus by a FluMist recipient in the same playgroup. This Type B isolate retained the *ca*, *ts*, and *att* phenotypes of the vaccine strain, and had the same genetic

sequence when compared to a Type B virus cultured from a vaccine recipient within the same playgroup. Four of the influenza Type A isolates were confirmed as wild-type A/Panama (H3N2). The remaining isolates could not be further characterized.

Assuming a single transmission event (isolation of the Type B vaccine strain), the probability of a young child acquiring vaccine virus following close contact with a single FluMist vaccinee in this daycare setting was 0.58% (95% CI: 0, 1.7) based on the Reed-Frost model. With documented transmission of one Type B in one placebo subject and possible transmission of Type A viruses in four placebo subjects, the probability of acquiring a transmitted vaccine virus was estimated to be 2.4% (95% CI: 0.13, 4.6), using the Reed-Frost model.

The duration of FluMist vaccine virus replication and shedding have not been established.

15 REFERENCES

1. Centers for Disease Control and Prevention. Prevention and Control of Influenza: Recommendations of the Advisory Committee on Immunization Practices (ACIP). *MMWR* 2007;56(RR-6):1-54.

16 HOW SUPPLIED/STORAGE AND HANDLING

FluMist is supplied for intranasal delivery in a package of 10 pre-filled, single-use sprayers. NDC 66019-106-01

Storage and Handling

Once FluMist has been administered, the sprayer should be disposed of according to the standard procedures for medical waste (e.g., sharps container or biohazard container).

FLUMIST SHOULD BE STORED IN A REFRIGERATOR BETWEEN 2-8°C (35-46°F) UPON RECEIPT AND UNTIL USE. THE PRODUCT MUST BE USED BEFORE THE EXPIRATION DATE ON THE SPRAYER LABEL.

DO NOT FREEZE.

The cold chain (2 to 8°C) must be maintained when transporting FluMist.

17 PATIENT COUNSELING INFORMATION

Vaccine recipients or their parents/guardians should be informed by the health care provider of the potential benefits and risks of FluMist, and the need for two doses at least 1 month apart in children 2-8 years old who have not previously received influenza vaccine.

17.1 Asthma and Recurrent Wheezing

Ask the vaccinee or their parent/guardian if the vaccinee has asthma. For children <5 years of age, also ask if the vaccinee has recurrent wheezing since this may be an asthma equivalent in this age group.

17.2 Vaccination with a Live Virus Vaccine

Vaccine recipients or their parents/guardians should be informed by the health care provider that FluMist is an attenuated live virus vaccine and has the potential for transmission to immunocompromised household contacts.

17.3 Adverse Event Reporting

The vaccine recipient or the parent/guardian accompanying the vaccine recipient should be told to report any suspected adverse events to the physician or clinic where the vaccine was administered.

FluMist® is a registered trademark of MedImmune, LLC.

MedImmune

Manufactured by:

MedImmune Vaccines, Inc.

Gaithersburg, MD 20878

1-800-633-4411

Issue Date: June 2008 RAL-FLUV8

U.S. Government License No. 1652

Appendix 2 Intranasal Sprayer Administration Instructions

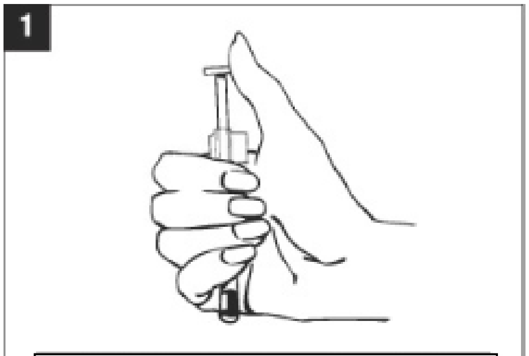

The intranasal spray applicator should be brought to room temperature just prior to administration by holding the sprayer in the palm of the hand, supporting the plunger rod with the thumb. Do not roll the sprayer or depress the plunger.

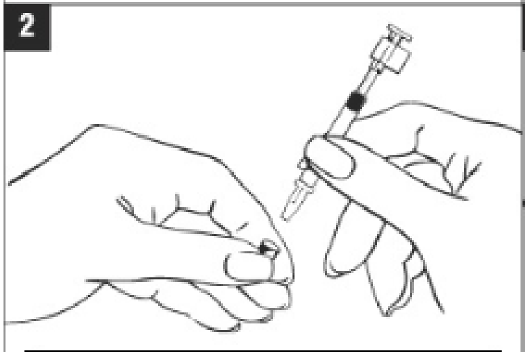

Remove rubber top protector.

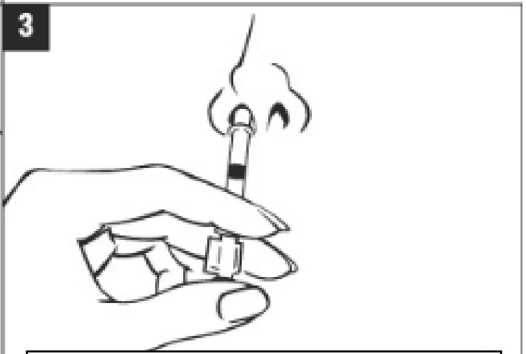

While the patient is in an upright position with head titled back, place the tip just inside the nostril to ensure the intranasal spray is delivered into the nose. Rapidly depress the plunger until the dose-divider clip prevents you from going further.

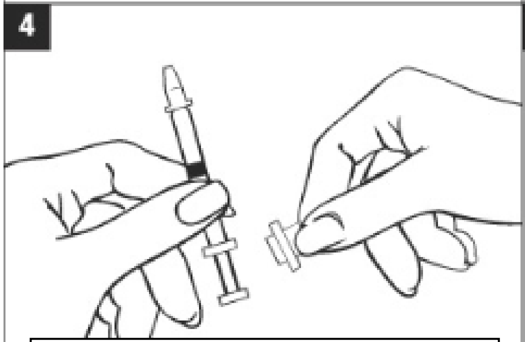

Pinch and remove dose-divider clip from plunger.

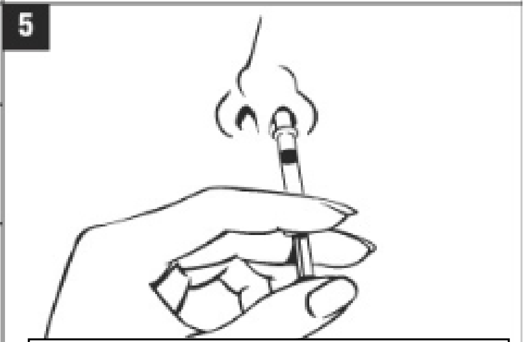

Place the tip just inside the other nostril and depress plunger to deliver remaining vaccine.
